# Enrichment of NPC1-deficient cells with the lipid LBPA stimulates autophagy, improves lysosomal function, and reduces cholesterol storage

**DOI:** 10.1016/j.jbc.2021.100813

**Published:** 2021-05-21

**Authors:** Olga Ilnytska, Kimberly Lai, Kirill Gorshkov, Mark L. Schultz, Bruce Nguyen Tran, Maciej Jeziorek, Thaddeus J. Kunkel, Ruth D. Azaria, Hayley S. McLoughlin, Miriam Waghalter, Yang Xu, Michael Schlame, Nihal Altan-Bonnet, Wei Zheng, Andrew P. Lieberman, Radek Dobrowolski, Judith Storch

**Affiliations:** 1 Department of Nutritional Sciences, Rutgers University, New Brunswick, New Jersey, USA; 2 Rutgers Center for Lipid Research, Rutgers University, New Brunswick, New Jersey, USA; 3 National Center for Advancing Translational Sciences, National Institutes of Health, Bethesda, Maryland, USA; 4 Department of Pathology, University of Michigan Medical School, Ann Arbor, Michigan, USA; 5 Department of Biological Sciences, Rutgers University, Newark, New Jersey, USA; 6 Department of Neurology, University of Michigan Medical School, Ann Arbor, Michigan, USA; 7 Departments of Anesthesiology and Cell Biology, New York University School of Medicine, New York, New York, USA; 8 Laboratory of Host-Pathogen Dynamics, National Heart, Lung and Blood Institute, Bethesda, Maryland, USA

**Keywords:** cholesterol, Niemann–Pick type C disease, autophagy, acid sphingomyelinase, lysobisphosphatidic acid, AC, acid ceramidase, AML, amitriptyline, ASM, acid sphingomyelinase, AP, autophagosome, BafA1, Bafilomycin A1, BMP, bis(monoacylglycerol)phosphate, CL, cardiolipin, DHA, docosahexaenoyl, EACC, ethyl (2-(5-nitrothiophene-2-carboxamido) thiophene-3-carbonyl) carbamate, ER, endoplasmic reticulum, FBS, fetal bovine serum, ILV, intralumenal vesicle, iPSC, induced pluripotent stem cell, LAMP1, lysosomal-associated membrane protein, LBPA, lysobisphosphatidic acid, LC3, microtubule-associated proteins 1A/1B light chain 3, LE, late endosome, LY, lysosome, MVB, multivesicular bodies, NPC1, Niemann–Pick type C1, NSC, neural stem cell, PC, phosphatidylcholine, PFO, perfringolysin O, PG, phosphatidylglycerol, PI, phosphatidylinositol, PM, plasma membrane, PUFA, polyunsaturated fatty acid, SM, sphingomyelin, *SMPD1*, gene encoding acid sphingomyelinase, SREBP2, sterol regulatory element binding protein 2

## Abstract

Niemann–Pick C (NPC) is an autosomal recessive disorder characterized by mutations in the *NPC1* or *NPC2* genes encoding endolysosomal lipid transport proteins, leading to cholesterol accumulation and autophagy dysfunction. We have previously shown that enrichment of NPC1-deficient cells with the anionic lipid lysobisphosphatidic acid (LBPA; also called bis(monoacylglycerol)phosphate) *via* treatment with its precursor phosphatidylglycerol (PG) results in a dramatic decrease in cholesterol storage. However, the mechanisms underlying this reduction are unknown. In the present study, we showed using biochemical and imaging approaches in both NPC1-deficient cellular models and an NPC1 mouse model that PG incubation/LBPA enrichment significantly improved the compromised autophagic flux associated with NPC1 disease, providing a route for NPC1-independent endolysosomal cholesterol mobilization. PG/LBPA enrichment specifically enhanced the late stages of autophagy, and effects were mediated by activation of the lysosomal enzyme acid sphingomyelinase. PG incubation also led to robust and specific increases in LBPA species with polyunsaturated acyl chains, potentially increasing the propensity for membrane fusion events, which are critical for late-stage autophagy progression. Finally, we demonstrated that PG/LBPA treatment efficiently cleared cholesterol and toxic protein aggregates in Purkinje neurons of the NPC1^I1061T^ mouse model. Collectively, these findings provide a mechanistic basis supporting cellular LBPA as a potential new target for therapeutic intervention in NPC disease.

Niemann–Pick type C disease is an autosomal recessive neurodegenerative disorder that is caused by loss of function mutations in the *NPC1* or *NPC2* genes (1, 2), which lead to aberrant trafficking of cholesterol through the endolysosomal system and secondary accumulation of glycosphingolipids (3).

The majority of LDL-derived unesterified (free) cholesterol in the late endosomal/lysosomal (LE/LY) compartment is found in inner membrane lamellae (or intraluminal vesicles, ILVs) within the lumen of the LE/LY (4, 5). The soluble NPC2 protein, localized within the interior of the LE/LY, binds cholesterol and transfers it to the luminal-facing N-terminal domain of the NPC1 protein localized to the limiting membrane of the compartment (6, 7, 8, 9). NPC1 protein, in turn, exports cholesterol out of the LE/LY for subsequent distribution to other compartments including recycling endosomes, the endoplasmic reticulum (ER), Golgi, mitochondria, and the plasma membrane (PM), and eventually for extracellular efflux from the cell (10, 11). In patients with NPC the egress of cholesterol from the LE/LY is dysfunctional. Cholesterol accumulation leads to an interruption in cellular cholesterol homeostasis and aberrant LE/LY function (3, 12), including defective autophagic flux, with impairment in the fusion of autophagosomes (APs) with LY to form autolysosomes. This results in the buildup of damaged cellular components and other debris and contributes to disease progression (13, 14, 15, 16, 17).

The ILV membranes where cholesterol accumulates are enriched in the atypical phospholipid lysobisphosphatidic acid (LBPA), also known as bis(monoacylglycero)phosphate (BMP) (18, 19, 20). LBPA accounts for approximately 15 to 20 Mol% of total phospholipids in the LE/LY and is not detected in any other subcellular compartments (18, 21). We showed that LBPA directly binds to NPC2 and markedly accelerates the cholesterol transport rates of NPC2 (22, 23, 24, 25). Recently, we demonstrated that LBPA enrichment of patient-derived NPC1-deficient fibroblasts *via* treatment with its presumed precursor phosphatidylglycerol (PG) (25, 26) led to a dramatic reduction in cholesterol storage (25). Direct supplementation with LBPA also reduced cholesterol accumulation in NPC1-deficient cells (27). Of note, PG/LBPA enrichment of NPC2-deficient cells was unable to induce cholesterol clearance (25). Thus, NPC2 and LBPA work together to traffic cholesterol through the LE/LY compartment *via* a process that can apparently bypass NPC1. However, the precise mechanism of this NPC2-dependent, NPC1-independent cholesterol egress from the endolysosomal compartment is unknown.

We recently showed that PG incubation of NPC1-deficient cells leads to an increase in exosome biogenesis and cholesterol secretion (28). In this report we demonstrate that PG/LBPA enrichment of NPC1-deficient cells also reverses the impaired fusion of AP with LY, resulting in an improvement of autophagic flux, and that this induction of autophagy also serves as a route for endolysosomal cholesterol efflux. The stimulation of autophagic maturation occurs, at least in part, through activation of acid sphingomyelinase, known to be suppressed in NPC1 cells (29, 30), as well as *via* the generation of membrane fusogenic LBPA species with polyunsaturated acyl chains. In addition, we present proof-of-concept data demonstrating LBPA induction of cholesterol clearance and autophagic flux in induced pluripotent stem cell (iPSC)-derived neuronal NPC1 cells and in an NPC1 animal model, supporting the therapeutic potential of PG/LBPA enrichment for treatment of the fatal neurodegenerative disorder NPC1.

## Results

### PG supplementation induces LBPA synthesis and endolysosomal cholesterol clearance in multiple NPC1-disease cell models

We previously reported that supplementation of primary fibroblasts from NPC1 patients with liposomes composed of 100% dioleoyl-PG resulted in LBPA enrichment and marked cholesterol egress from endolysosomal compartments (25). To further explore the mechanism of PG/LBPA action, we first complemented our studies in the GM03123 human NPC1-deficient dermal fibroblast line (25) by using CRISPR-Cas9-edited NPC1 knockout (KO) HeLa cells. The GM03123 line is a compound heterozygote harboring a P237S mutation in one allele and a second allele with an I1061T mutation (Coriell Cell Repository); thus, the HeLa KO cells allowed us to rule out a potential role of residual NPC1 protein in the NPC1-mutant fibroblasts. We also examined differentiated neural stem cells (NSCs) generated from the GM03123 NPC1-mutant fibroblasts *via* iPSC reprograming (31) to ensure that results in NPC1 fibroblasts were not cell specific, with the NSC model relevant for the neurodegenerative NPC1 disorder.

**Figure 1:**
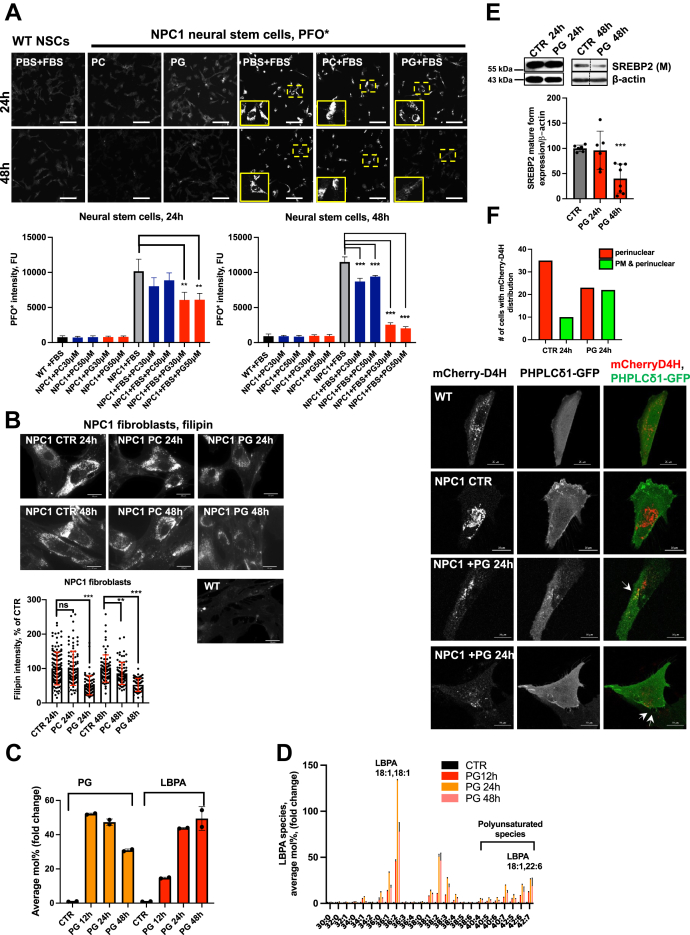
**PG treatment in NPC1 cellular models raises LBPA levels and restores cholesterol homeostasis.***A*, representative PFO∗ images of differentiated WT (GM05659) and NPC1 (GM03123) fibroblasts to neural stem cells (NSC) and treated with 30 or 50 μM of PC and PG for 24 and 48 h in the absence and presence of 2.5% FBS to induce cholesterol accumulation. Bar graph represents quantification of PFO∗ intensity. Scale bar, 50 μm. N = 200 to 600 cells/condition. *B*, representative epifluorescent images and quantification of filipin intensity in WT or NPC1 mutant human fibroblasts treated with 100 μM PG or PC for 24 and 48 h. Scale bar, 30 μm. Dots represent fluorescent intensities of single cells. Data from three independent experiments. N = 78 to 125 cells/condition. *C*, LC-MS analysis of total PG and LBPA levels in NPC1-mutant fibroblasts treated with 100 μM PG for 12, 24, and 48 h. N = 2. See also Fig. S1, *C*–*E*. *D*, LC-MS analysis of molecular species of LBPA in NPC1-deficient fibroblasts treated with 100 μM PG for 12, 24, and 48 h relative to untreated control. *E*, Western blot analysis of expression of mature form of SREBP2 at 24 and 48 h of PG treatment in NPC1-mutant fibroblasts. On the *right panel* samples were analyzed in biological replicates. For each condition one band was cropped and bands for CTR and PG were spliced. Splicing is indicated with a vertical line. PG concentration 100 μM. Data from three independent experiments. N = 6 to 8. *F*, mCherry-D4H cholesterol reporter distribution in live NPC1 mutant fibroblasts treated with 100 μM PG or vehicle control for 24 h. Total N = 40 cells/group expressing reporter were imaged using an epifluorescent microscope and analyzed for reporter distribution pattern. Representative confocal images of WT and NPC1 mutant fibroblasts coexpressing mCherry-D4H and PH-PLCδ1-GFP show partial colocalization of reporters in PG-treated cells. Scale bar, 20 μm. All graphed data show mean ± SD. ∗∗*p* < 0.01, ∗∗∗*p* < 0.001 compared with untreated cells (CTR) in two-tailed *t* test. FBS, fetal bovine serum; LBPA, lysobisphosphatidic acid; NPC1, Niemann–Pick type C1; PC, phosphatidylcholine; PG, phosphatidylglycerol.

To induce the accumulation of LDL-derived cholesterol in the endolysosomal compartment of NPC1-mutant NSCs we grew cells in the presence of fetal bovine serum (FBS) for 24 h prior to liposome treatment. We initially titrated the FBS concentration and established an optimal 2.5% concentration, since higher concentrations of FBS significantly diminished cell viability (not shown). In contrast to WT cells and in agreement with previous studies (31), NPC1 NSCs accumulated LDL cholesterol in the LE/LY compartment (Fig. 1*A*). After 24 h culturing in the presence of FBS, cells were treated with 100 mol% PG or phosphatidylcholine (PC) liposomes for an additional 24 or 48 h. Unesterified cholesterol levels in NPC1 mutant fibroblasts and NPC1 KO HeLa cells were determined quantitatively using image analysis of the fluorescent antibiotic filipin or the fluorescently labeled cytolysin PFO∗, as previously described (25, 32).

PG treatment resulted in significant correction of the NPC1 phenotype in all three cellular model systems at 24 and 48 h, as observed by reduced filipin and PFO∗ intensities (Fig. 1, *A* and *B* and Fig. S1*A*). In marked contrast, supplementation with PC had no effect on LE/LY cholesterol storage at 24 h and showed a small reduction at 48 h, indicating that the effect of cholesterol clearance is PG specific.

LC-MS analysis of phospholipids in PG-treated NPC1-deficient fibroblasts at 12, 24, and 48 h post treatment revealed remarkable increases of up to 50-fold in both PG and LBPA levels in NPC1 cells (Fig. 1*C*). Phosphatidylinositol (PI) and *N*-acyl phosphatidylethanolamine increased approximately 2.5-fold, and other phospholipid levels, except acyl-PG, were unchanged (Fig. S1*B*). PG levels declined over time, whereas LBPA levels increased, further indicating conversion of PG to LBPA. The gradual declines in *N*-acyl phosphatidylethanolamine and acyl-PG suggest they may be synthetic intermediates, although this remains to be determined. Of interest, the analysis of LBPA molecular species in NPC1 fibroblasts treated with di-oleoyl PG revealed not only an expected increase in di-oleoyl LBPA but also marked, up to 25-fold increases in LBPA species incorporating polyunsaturated fatty acids (PUFAs), *e.g.*, 18:1,22:6-LBPA (oleoyl, docosahexaenoyl (DHA)-LBPA) (40:7 in Fig. 1*D*), relative to untreated cells.

We noted that NPC1-deficient fibroblasts supplemented with PC showed an approximately 2-fold increase in PG, and LBPA levels were elevated by approximately 50% (Fig. S1*C*). Exogenous PC can serve as a substrate for PG synthesis *via* a transphosphatidylation reaction (33); thus, these relatively small increases in PG and LBPA might explain the slight cholesterol reduction in all PC-treated NPC1 cellular models after 48 h (Fig. 1, *A* and *B* and Fig. S1*A*). Overall, these results indicate a specific effect of PG/LBPA enrichment on cholesterol clearance.

PG is also the precursor of another atypical phospholipid, cardiolipin (CL) (34). LC-MS analysis of CL levels in NPC1-deficient cells revealed no change over 48 h of PG treatment (Fig. S1*D*), indicating that the exogenous PG is not utilized for CL biosynthesis, but rather for LBPA biosynthesis, in NPC1 cells. This may be due to introduction of the precursor to the endosomal vesicle system, separate from subcellular sties of CL generation, as well as the understanding that CL concentrations are tightly controlled by its abundance in mitochondria (35). Of importance, PG supplementation does not affect cell viability in NPC1-deficient cells even when added for up to 6 days (Fig. S1*E*).

We next investigated whether PG treatment in NPC1-deficient fibroblasts led to endolysosomal cholesterol redistribution to the ER compartment. Increased cholesterol levels in the ER repress processing of the master transcription factor sterol regulatory element binding protein 2 (SREBP2) into its mature, transcriptionally active form ((M)SREBP2) (36). Of interest, despite significant LE/LY cholesterol reduction at 24 h of PG treatment, the level of (M)SREBP2 was not changed (Fig. 1*E*), implying that cholesterol did not reach the regulatory pool in the ER at this time point. By contrast, at 48 h following PG treatment the (M)SREBP2 level was decreased by approximately 50%, suggesting delivery of cholesterol to the ER.

Egress of cholesterol from the LE/LY is reportedly followed by its residence in the PM (11, 37). To identify cholesterol at the cytoplasmic leaflet of the PM in live cells, we used a perfringolysin O (PFO)-derived cholesterol-binding probe, mCherry-D4^D434S^ mutant (named D4H) (38). In WT fibroblasts we found that the mCherry-D4H reporter was mainly localized to the cytosolic leaflet of the PM and to recycling endosomes enriched in cholesterol (Fig. 1*F*). NPC1-deficient cells have been shown to have a reduced amount of cholesterol in the PM (39). Consistent with this, we found that, in untreated NPC1 cells, the D4H reporter was more heavily localized to the abundant perinuclear cholesterol-enriched endolysosomal compartments than to the PM, with only ∼25% of cells expressing the D4H reporter showing both perinuclear and peripheral PM signal (Fig. 1*F*). In contrast, we observed that, at 24 h of PG treatment in NPC1-deficient cells, D4H was localized to the PM in ∼50% of cells, where it colocalized with a PLC*δ*-PH domain fused to GFP (PHPLC*δ*-GFP), a phosphatidylinositol-4,5-bisphosphate (PI(4,5)P_2_) probe and PM marker (40) (Fig. 1*F*).

Together these data suggest the trafficking of endolysosomal cholesterol to the PM at earlier (≤ 24 h) time points and to the ER at later (>24 h) time points of PG treatment, indicating the restoration of normal cholesterol trafficking and distribution.

### LBPA enrichment re-establishes lysosomal homeostasis and autophagic flux in NPC1-deficient cells

**Figure 2:**
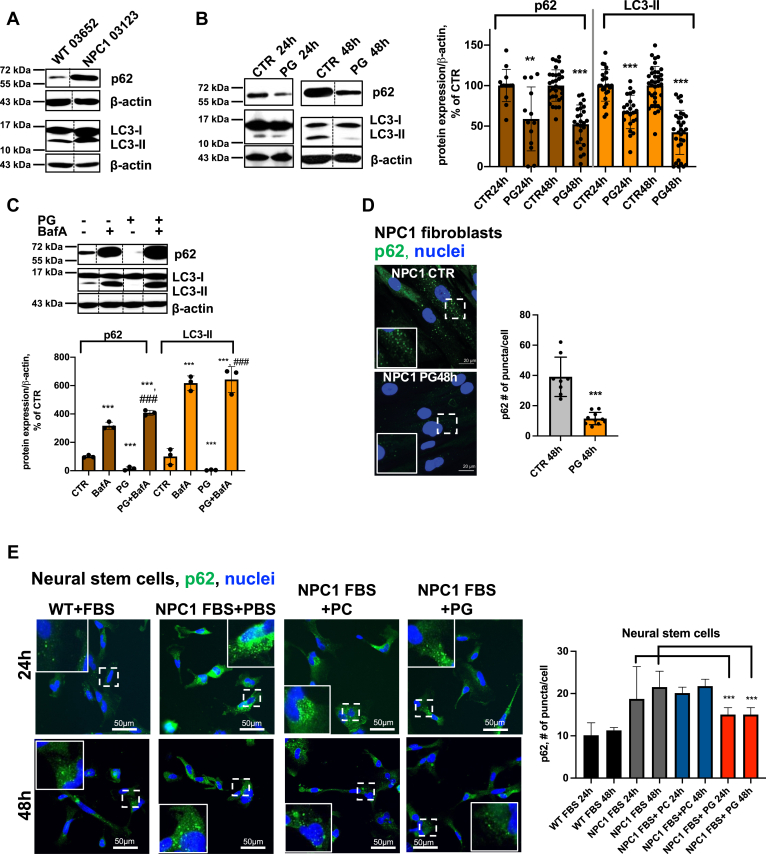
**PG/LBPA enrichment increases autophagic clearance in NPC1-deficient fibroblasts**. *A*, Western blot analysis of LC3-II and p62 in WT and NPC1 fibroblasts. *B*, Western blot analysis and quantification of LC3-II and p62 in NPC1 fibroblasts treated with 100 μM PG for 24 and 48 h relative to untreated CTR. Data from 6 to 8 independent experiments. *C*, Western blot analysis shows inhibition of LC3-II and p62 clearance by BafA1. Cells were treated with 100 μM PG for 48 h, and BafA1 (100 nM) was added for the last 24 h. In *A*–*C* (images) all conditions were analyzed in biological replicates. For each condition one band was cropped and bands for all conditions were spliced. Splicing is indicated with a *vertical line*. *D*, confocal images and quantification of p62-positive puncta in NPC1 fibroblasts at 48 h. Scale bar, 20 μm. Individual *dots* represent average # per cell/image. Average cell N in image is 2 to 6 cells. *E*, fluorescent staining and quantification of p62- positive puncta in WT and NPC1-deficient neural stem cells. Scale bar, 50 μm. N ≥ 2000 cells/condition. PG 50 μM. All graphed data show mean ± SD. ∗∗*p* < 0.01, ∗∗∗*p* < 0.001 compared with untreated cells (CTR), ^###^*p* < 0.001 compared with PG treated in two-tailed *t* test. NPC1, Niemann–Pick type C1; PG, phosphatidylglycerol.

Defective autophagy is implicated in the pathogenesis of many neurodegenerative and lysosomal storage diseases including NPC1, where impaired autophagosome–lysosome (AP-LY) fusion has been reported (13, 14, 17). Since LBPA has membrane fusogenic properties (19, 20, 41), we investigated whether PG-induced LBPA enrichment alters autophagic clearance in NPC1 patient fibroblasts. Consistent with previous reports (14, 42) we found that the levels of two autophagic protein markers, the AP-associated form of lipidated microtubule associated protein 1 light-chain 3 (LC3-II) and a selective substrate of the autophagy degrading pathway, polyubiquitin binding protein p62/SQSTM1 (p62), were significantly higher in NPC1 fibroblasts compared with WT fibroblasts, indicating increased levels of AP and their failed breakdown due to impaired autophagic flux (Fig. 2*A*). Treatment with PG gradually reduced the levels of LC3-II and p62 over 24 to 48 h, suggesting an induction of autophagic protein degradation in NPC1-mutant fibroblasts and NPC1 KO HeLa cells (Fig. 2*B* and Fig. S2*A*). The PG-induced p62 and LC3-II protein reduction was abrogated in the presence of Bafilomycin A1 (BafA1) (Fig. 2*C*), an inhibitor of LY function and of the late phase of autophagy (43), indicating specific LY degradation of p62 and LC3-II.

Both the number and size of p62- and LC3-positive puncta (shown in insets), indicative of AP, were consistently reduced in PG-treated NPC1 fibroblasts, NPC1-mutant NSC cells, and NPC1 KO HeLa cells (Fig. 2, *D* and *E* and Fig. S2, *B*–*D*). Upstream events regulating AP biogenesis, including the mTOR signaling pathway and its downstream serine/threonine kinase p70S6K, AMPK activity, as well as beclin-1 expression levels, were not altered in PG-treated NPC1 fibroblasts (Fig. S2*E*). Taken together, the results indicate that PG/LBPA enrichment specifically impacts autophagic maturation.

**Figure 3:**
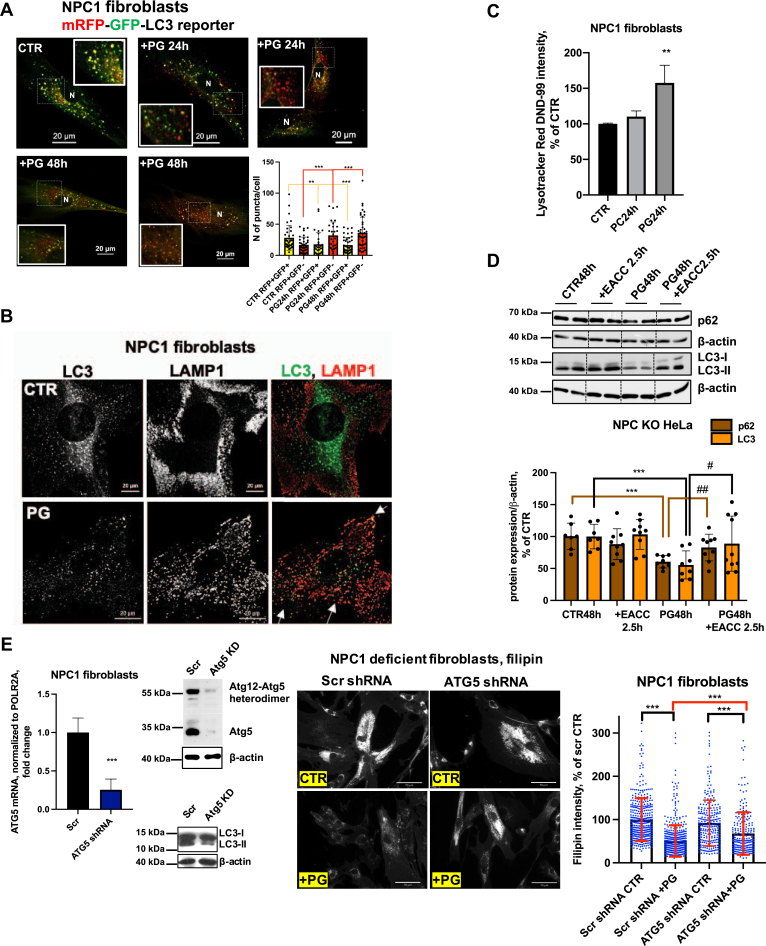
**PG/lysobisphosphatidic acid enrichment increases the formation of autolysosomes, reduces lysosomal clustering, improves lysosomal mobility, and induces cholesterol efflux in autophagy-dependent manner.***A*, PG treatment for 24 h improves the impaired autophagic flux by facilitating formation of autolysosomes (*red puncta*) and reducing the number of autophagosomes (*yellow puncta*). Representative confocal images and image quantification from four independent experiments. N ≥ 30 cells/condition. *B*, confocal images of LAMP1 and LC3 in NPC1 fibroblasts treated with 100 μM PG for 48 h. Scale bar, 20 μm. *Arrows* indicate redistribution and colocalization of LC3 and LAMP1 in PG-treated cells. *C*, flow cytometry analysis of Lysotracker Red DND-99 intensity. N = 3 biological replicates/condition, each sample contained 4000 cells. *D*, Western blot analysis of p62 and LC3-II expression in NPC1 KO HeLa cells treated with PG for 48 h, PG for 46.5 h+ EACC for 2.5 h (10 μM), EACC alone for the last 2.5 h of the experiment. On the *upper panel* all conditions were analyzed in biological triplicates. For each condition two bands were cropped and bands for all conditions were spliced. Splicing is indicated with a *vertical line*. Data from three independent experiments. *E*, mRNA expression and Western blot analysis of Atg5 and Atg12-Atg5 heterodimer expression, LC3 expression, filipin staining and quantification of filipin intensity in NPC1-deficient fibroblasts stably transduced with lentiviral nonsilencing (Scr) or Atg5 shRNA and selected for stable shRNA expression. N ≥ 220 cells/condition. Data from two independent experiments. Scale bar, 70 μm All graphed data show mean ± SD. ∗∗*p* < 0.01, ∗∗∗*p* < 0.001 compared with untreated cells (CTR, Scr), ^#^*p* < 0.05, ^##^*p* < 0.01 compared with PG in two-tailed *t* test. NPC1, Niemann–Pick type C1; PG, phosphatidylglycerol.

To further examine the effects of PG/LBPA enrichment on autophagic flux in NPC1-deficient fibroblasts, we employed mRFP-GFP-LC3-II tandem sensor to monitor fusion of AP and LY to form autolysosomes. This sensor consists of an acid-sensitive GFP, which is quenched at acidic pH, and an acid-resistant RFP, which sustains its fluorescent emission in acidic pH, permitting the progression from the AP with neutral pH to acidified AL to be monitored (44). In NPC1-deficient cells expressing mRFP-GFP-LC3-II, yellow puncta (mRFP+-GFP+) indicate AP, whereas the red puncta (mRFP+-GFP-) indicate AL (44) (Fig. 3*A*). Untreated cells are seen to have only a few red puncta, in keeping with the known impairment in AP-LY fusion. PG treatment for 24 and 48 h reduces the yellow puncta and increases the red puncta, indicating an increase in AL formation, thus allowing degradation of the enclosed proteins and lipids.

To directly analyze AP-LY fusion, we examined colocalization between the endogenous LY marker lysosomal-associated membrane protein 1 (LAMP1) and the AP marker LC3 in NPC1-deficient fibroblasts. We found that, in untreated NPC1-mutant fibroblasts, the LAMP1-positive LY and LC3-positive AP were separately distributed. Following PG treatment, however, these organelles were redistributed, appeared in closed proximity, and, despite the reduced amount of LC3-positive AP, colocalized to a greater extent (Fig. 3*B*). The time course showing immunofluorescent staining of another major LY marker, LAMP2, revealed reductions in abnormal LY size (shown in insets) and LY clustering, both of which are hallmarks of NPC1 disease, in PG-treated cells (Fig. S2*F*). Thus, the PG treatment resulted in reduction of LY size and clustering, an increase in their motility, and a redistribution away from the perinuclear area where they accumulate in untreated cells.

We then investigated whether PG treatment increases lysosomal acidification using LysoTracker Red DND-99, a cell permeable weak base dye that is acidotrophic and accumulates in acidified organelles (45). Lysosomal pH in NPC1 disease has been reported as elevated compared with healthy controls (46). Flow cytometry analysis revealed a significant increase of LysoTracker intensity in PG-treated cells at 24 h, but not in PC-treated cells (Fig. 3*C*), suggesting a PG-specific reacidification of LY and improvement of lysosomal homeostasis.

Since the restoration of acidic pH in the LY compartment might have contributed to the GFP quenching of the LC3-II reporter shown in Figure 3*A*, we investigated the effect of perturbing AP-LY fusion on PG-induced autophagic clearance using the small molecule inhibitor ethyl (2-(5-nitrothiophene-2-carboxamido) thiophene-3-carbonyl) carbamate (EACC), which selectively blocks the late stage of autophagy (AP-LY fusion) by inhibiting STX17 translocation onto autophagosomes and does not affect LY pH and function (47). The inhibitor was added acutely for only 2.5 h after 46.5 h of PG treatment in NPC1 KO HeLa cells. This compound effectively blocked PG-induced p62 and LC3-II clearance (Fig. 3*D*), indicating that the clearance is mediated by AP-LY fusion.

We hypothesized that enhanced formation of AP can provide a route for cholesterol clearance from the LE/LY compartment. To test this hypothesis and to determine whether PG/LBPA-induced autophagic flux and cholesterol clearance were directly linked we evaluated the efficacy of PG treatment on cholesterol clearance in autophagy-impaired Atg5-depleted NPC1-deficient fibroblasts. Core autophagy protein Atg5 conjugates with Atg12 to form an Atg12–Atg5 complex that is essential for formation of AP and autophagy progression (48). Atg5 depletion using shRNA resulted in significant (∼75%–85%) reductions in Atg5 mRNA and protein expression and in Atg5–Atg12 protein conjugates in NPC1 fibroblasts (Fig. 3*E*). The level of LC3-II, as expected, was significantly reduced in Atg5 knockdown cells, indicating a reduction of AP formation (Fig. 3*E*). We found that the PG effect on cholesterol clearance was significantly but only modestly attenuated in Atg5-depleted compared with scrambled control cells, by ∼23%. An incomplete blocking of PG effectiveness in Atg5 knockdown cells is likely due to the presence of residual Atg5; however, it also suggests the existence of additional routes of cholesterol mobilization. Indeed, we recently found that cholesterol egress *via* exosome release is upregulated in PG/LBPA-enriched NPC1 cells (28).

These data indicate that autophagic flux contributes to the egress of cholesterol from the late endosomal compartment and suggest that the impaired ability of LY to undergo efficient fusion with AP was restored by PG/LBPA enrichment in NPC1-deficient cells. The luminal domains of NPC1 are thought to be required for cholesterol to penetrate through the glycocalyx that coats the luminal leaflet of the limiting LY membrane (6, 49); thus, enhanced AP-LY fusion could potentially provide a route for cholesterol egress from the LE/LY that bypasses the glycocalyx.

Overall, these data show that PG treatment results in the reacidification of LY, a reduction in LY size, increased LY motility, and increased fusion with AP.

### PG/LBPA enrichment increases acid sphingomyelinase activity

We next sought to understand the mechanism by which LBPA enrichment might enhance autophagic flux. Acid sphingomyelinase (ASM) is known to be impaired in NPC1 cells (29, 30), and *in vitro* liposome-based studies showed that LBPA binding to ASM facilitates its activity (50, 51). In addition, ASM and its product ceramide appear to play regulatory roles in autophagic flux (52, 53, 54, 55). Thus, we hypothesized that the mechanism by which LBPA enrichment stimulates the late stages of autophagy in NPC1 cells may be *via* modulation of ASM activity.

**Figure 4:**
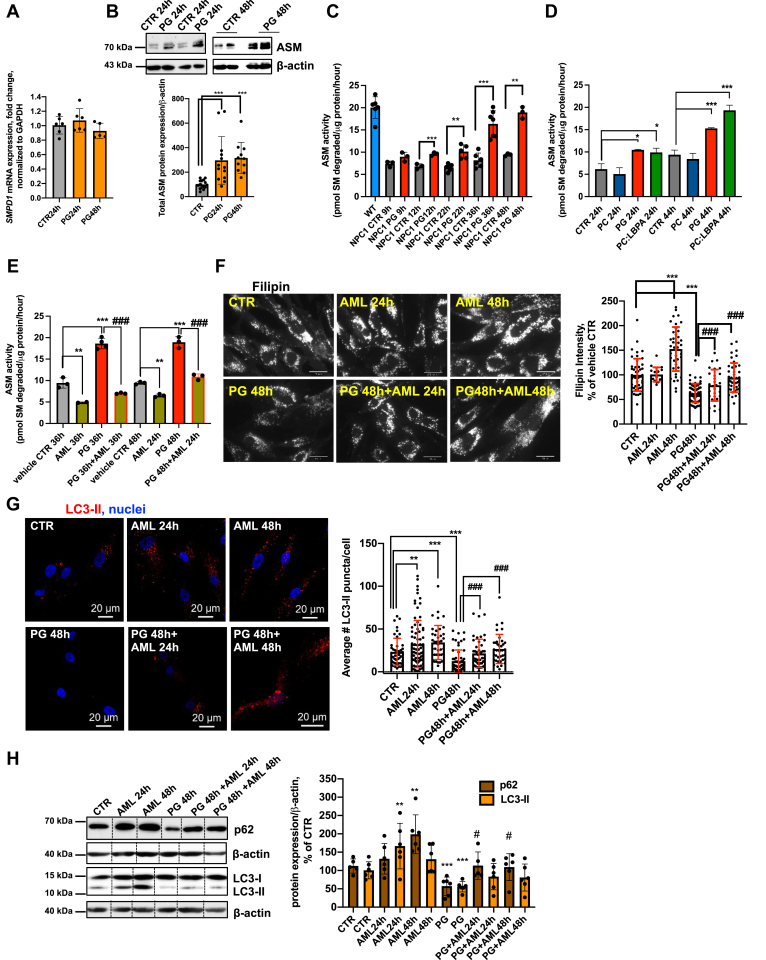
**ASM regulation of cholesterol clearance and autophagic flux in PG-treated NPC1-deficient fibroblasts.***A*, time course of mRNA expression of *SMPD1* in PG-treated NPC1-deficient fibroblasts. (PG-100 μM). *B*, time course of ASM protein expression in PG-treated NPC1-deficient fibroblasts (PG-100 μM). *C*, ASM activity in WT and NPC1-deficient fibroblasts, treated with PG (100 μM) for 9 to 48 h. *D*, ASM activity in NPC1 fibroblasts treated with PG (100 μM), PC (100 μM), and PC:LBPA (1:1 mol:mol) (100 μM:100 μM) for 44 h. *E*, the ASM inhibitor amitriptyline (AML) (5 μM) significantly inhibits ASM activity. *F*, AML, 5 μM, increases cholesterol levels in control NPC1 fibroblasts and diminishes the cholesterol clearance in PG-treated cells. Cells were treated with AML alone for 24 or 48 h, PG alone for 48 h, or PG for 48 h with AML for 24 or 48 h. Data from three independent experiments. Representative epifluorescent images of NPC1-deficient cells. Scale bar, 30 μm. N ≥ 80 cells/condition. *G*, AML increases accumulation of LC3-II in untreated NPC1 cells and diminishes the stimulatory effect of PG on LC3-II degradation in treated cells. Cells were treated with AML as in *F*. Data from two independent experiments. Representative confocal images. Scale bar, 20um. N ≥ 80 cells/condition. *H*, Western blot analysis of p62 and LC3-II in NPC1 fibroblasts treated with PG, AML alone, or PG+AML as in *F* and *E*. On the *upper panel* all conditions were analyzed in biological replicates. For each condition one band was cropped and bands for all conditions were spliced. Splicing is indicated with a *vertical line*. All graphed data show mean ± SD. ∗*p* < 0.05, ∗∗*p* < 0.01, ∗∗∗*p* < 0.001 compared with control, ^#^*p* < 0.05, ^###^*p* < 0.001 compared with PG in two-tailed Student’s test. ASM, acid sphingomyelinase; NPC1, Niemann–Pick type C1; PG, phosphatidylglycerol.

We first tested whether PG/LBPA enrichment affected the expression of ASM and found that, while the mRNA encoding ASM, *SMPD1*, was unaffected by this treatment over 48 h (Fig. 4*A*), total ASM protein expression increased significantly, with evident processing of the 75-kDa ASM proprotein into the mature 70-kDa form (56) (Fig. 4*B*). We then tested whether PG or LBPA treatment increases ASM activity in NPC1 cellular models and found that PG and LBPA-treated NPC1-deficient fibroblasts (Fig. 4, *C* and *D*) and NPC1 KO HeLa cells (Fig. S3*A*) reached WT levels of ASM activity by 48 h of treatment; the increased activity correlated well with protein expression. The time-dependent increase in ASM activity also correlated well with the increased LBPA levels shown in Figure 1*C*. Of importance, PC-treated cells did not exhibit increased ASM activity, again underscoring a specific effect of PG/LBPA. Thus, PG/LBPA enrichment increases ASM protein maturation and its activity but not its mRNA.

To address whether PG-induced ASM activation was required for the associated cholesterol clearance and autophagic flux we used the cationic amphiphilic drug amitriptyline (AML), a functional ASM inhibitor. Cells were incubated with PG and AML simultaneously, and as shown in Figure 4*E*, AML dramatically suppressed PG stimulation of ASM activity in NPC1 fibroblasts and NPC1 KO HeLa cells (Fig. S3*B*). Even when AML was added after 24 h of PG treatment, a marked decrease in ASM activity was observed (Fig. 4*E*). The inhibition of ASM with AML abrogated the effect of PG/LBPA enrichment on cholesterol clearance (Fig. 4*F* and Fig. S3*C*).

AML treatment also markedly attenuated the stimulatory effects of PG on autophagic flux in NPC1 cellular models (Fig. 4, *G* and *H* and Fig. S3, *D* and *E*), indicating the involvement of ASM in PG/LBPA-stimulated autophagy. These data also support the notion that cholesterol clearance and autophagic flux are related processes in NPC1 cells.

We also considered that AML is not exclusively an inhibitor of ASM and might additionally affect acid ceramidase (AC) (57), an enzyme that metabolizes ceramide to sphingosine. Therefore, we used a specific inhibitor of AC, Bodipy-Soclac (58), to compare its effect with AML on cholesterol clearance in NPC1-deficient fibroblasts. Since the concentration of Bodipy-Soclac had not been established for long-term cell treatment, we tested a range of concentrations from 100 nM to 1 μM for 48 h of treatment. We showed that Bodipy-Soclac in concentrations of 100 nM to 1 μM effectively inhibited AC activity without affecting cell viability (Fig. S3, *F* and *H*). In contrast with AML, Bodipy-Soclac did not abrogate the PG effect on cholesterol clearance (Fig. S3*G*). Of note, the activity of AC, in contrast with ASM, was not upregulated by PG treatment (Fig. S3*F*). Overall, the data highlight a specific effect of ASM on cholesterol clearance.

**Figure 5:**
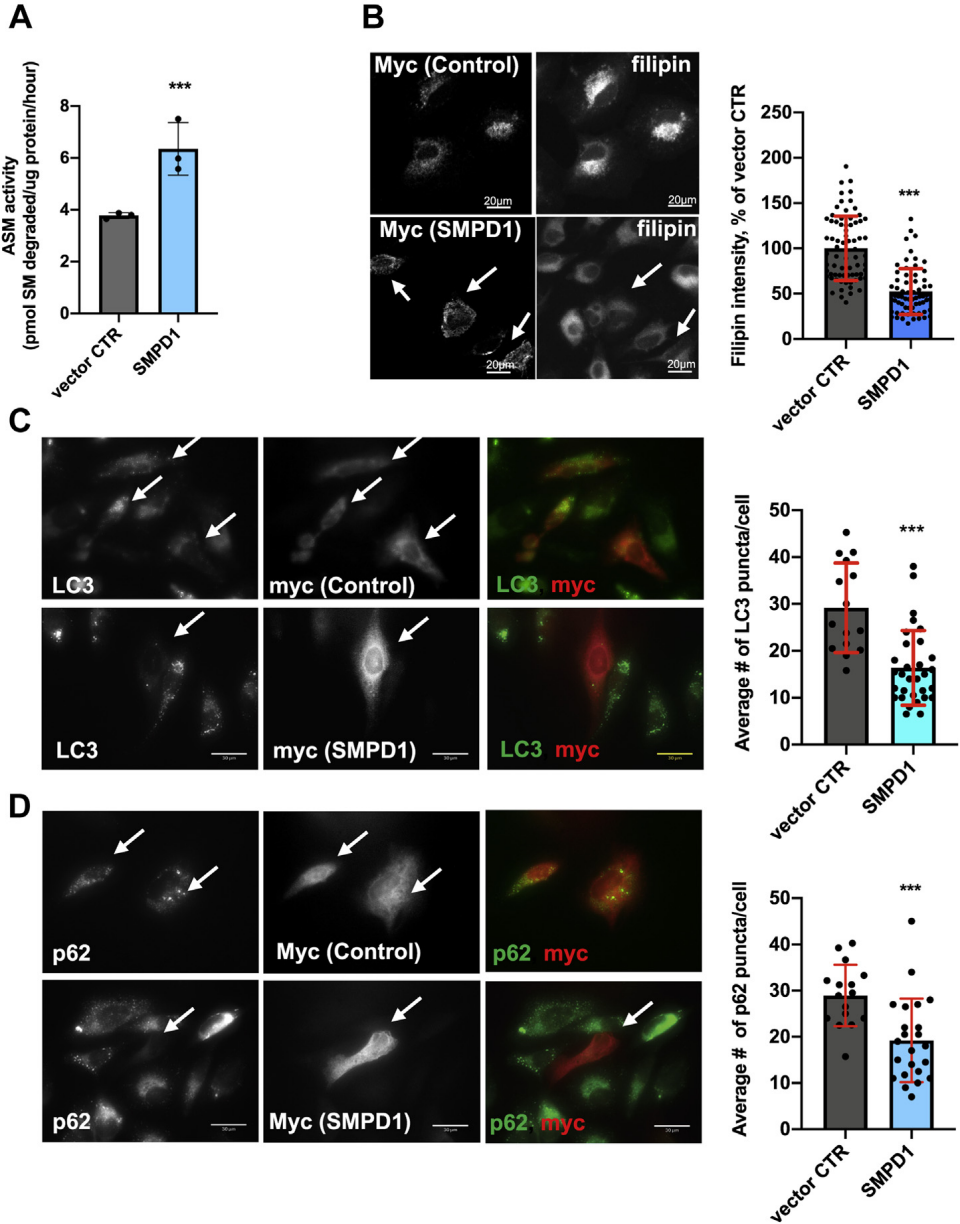
**ASM overexpression reduces cholesterol accumulation and increases autophagic flux in NPC1 KO HeLa cells.***A*, ASM activity in HeLa NPC1 KO cells transfected with empty vector (myc-tag) or *SMPD1*-myc at 24 h post transfection. *B*, representative confocal images and filipin intensity quantification in empty vector or *SMPD1*-transfected HeLa NPC1 KO cells at 24 h post transfection. N ≥ 70 cells/condition. *C*, representative epifluorescent images and quantification of number of LC3 puncta in NPC1 KO cells expressing vector CTR or *SMPD1* at 24 h post transfection. N ≥ 55 cells/condition. *D*, representative epifluorescent images and quantification of p62 puncta in NPC1 KO cells expressing vector CTR or *SMPD1* at 24 h post transfection. N ≥ 35 cells/condition. All graphed data show mean ± SD. ∗∗∗*p* < 0.001 *versus* vector control in two-tailed Student’s test. In all images, *arrows* indicate vector control myc-tag or *SMPD1*-myc tag-expressing cells. ASM, acid sphingomyelinase; NPC1, Niemann–Pick type C1.

Previous studies have shown that restoration of ASM activity by overexpression of *SMPD1* (ASM) in NPC1-deficient CHO cells and primary human fibroblasts resulted in marked reduction of endolysosomal cholesterol accumulation (29). To further explore the relationship between cholesterol clearance, autophagy, and ASM activity, we used a genetic restoration approach to investigate whether increased ASM activity affects autophagic flux. As a model, we used NPC1 KO HeLa cells transiently overexpressing *SMPD1* cDNA, since these cells have better transfection efficacy. In agreement with Devlin *et al.* (29) we found significant endolysosomal cholesterol reduction in *SMPD1*-overexpressing cells with increased ASM activity (Fig. 5, *A* and *B*). Furthermore, and corresponding with the results obtained in PG-treated cells, increased ASM activity resulted in LC3-II and p62 reduction (Fig. 5, *C* and *D*).

Collectively, these results suggest that PG/LBPA enrichment results in a restoration of ASM activity in NPC1 disease cellular models to the normal level found in healthy (WT) cells, which correlates with endolysosomal cholesterol clearance and autophagic flux enhancement. It should be noted that, to date, the stimulation of ASM activity by anionic lipids was shown only in reconstituted systems using liposomes and recombinant enzyme (51). Our data for the first time show that PG/LBPA treatment increases endogenous ASM activity in NPC1 disease cells.

### PG treatment clears cholesterol and stimulates autophagy in Purkinje neurons of Niemann–Pick C transgenic mice

**Figure 6:**
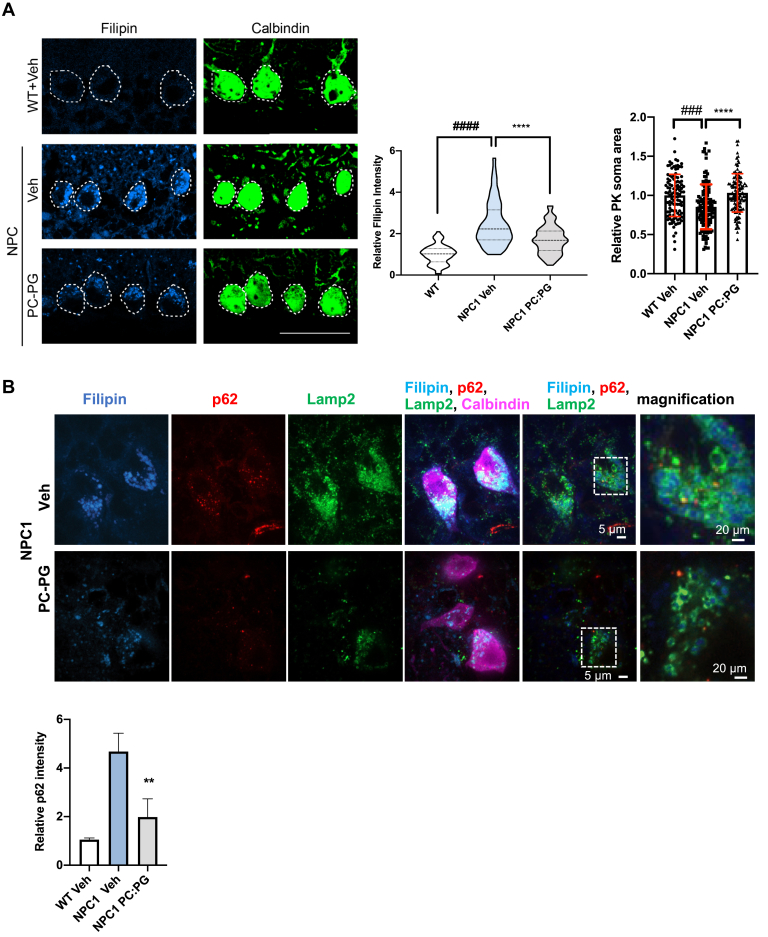
**PG treatment results in cholesterol and p62 clearance in Purkinje neurons of NPC1 *(I1061T/I1061T)* mice.***A*, cholesterol levels (filipin) and soma size in Purkinje neurons (calbindin) of WT and NPC1 *(I1061T/I1061T) mice* at 1 week post intracerebroventricular injections of PBS (Veh) or 800 μM PC:PG (1:1 mol:mol; 400 μM PC + 400 μM PG). Violin plot shows median (*dashed line*), 25% and 75% (*dotted lines*), and probability density (*thickness*). *Dashed lines* indicate Purkinje cell soma. Scale bar, 50 μm. Data are mean ± SD from n = 5 mice per treatment group, and cell numbers: WT+Veh = 110, NPC+Veh = 112, NPC+PC:PG = 122 cells. ∗∗∗∗*p* ≤ 0.0001: NPC1 vehicle treated compared with PC:PG treated, ^###^*p* ≤ 0.001, ^####^*p* ≤ 0.0001: NPC1 vehicle treated *versus* WT. One-way ANOVA with Tukey post hoc test (F, 101.9 df = (2)). *B*, p62 costaining with filipin, calbindin, LAMP2, and p62 quantification in calbindin-positive neurons of WT+Veh-, NPC1+Veh-, and NPC1+PC:PG-treated mice. ∗∗*p* ≤ 0.01. Scale bar, 5 μm. See also Fig. S4. NPC1, Niemann–Pick type C1; PC, phosphatidylcholine; PG, phosphatidylglycerol.

Based on the significant rescue of the NPC1 phenotype observed *in vitro*, we sought to determine whether administration of PG liposomes may benefit gene-targeted mice homozygous for the NPC1I1061T allele. I1061T is the most prevalent NPC1 disease-causing mutation (59). NPC1^I1061T/I1061T^ mice develop robust, progressive phenotypes including intracellular cholesterol accumulation, Purkinje neuron loss, motor impairment, and premature death by 13 weeks of age (59). Seven-week-old WT and NPC1^I1061T/I1061T^ mice received a single intracerebroventricular injection with vehicle (PBS) or 800 μM PC:PG (1:1 mol:mol; 400 μM PC + 400 μM PG). This composition and concentration of liposomes was established based on cell viability (Fig. S4*A*) and cholesterol clearance (Fig. S4*B*) studies in NPC1 fibroblasts. One week post injection, we calculated soma size in Purkinje neurons of the cerebellum that are especially sensitive to the loss of NPC1 function (60), as a potential indicator of *in vivo* toxicity of the PC:PG treatment and found that PC:PG treatment resulted in soma size similar to WT animals and in contrast to the classical shrunken neurons seen in vehicle-treated NPC1 animals (Fig. 6*A*).

Cholesterol levels were assessed by filipin staining. Remarkably, Purkinje neurons labeled with a specific marker, calbindin, showed a significant 30% reduction in cholesterol levels in the PC:PG-treated mice 7 days after a single injection (Fig. 6*A*).

Abnormal autophagy has been implicated as the cause of Purkinje cell death (60, 61), and our findings in NPC1-deficient cells, described above, indicate that PG/LBPA leads to cholesterol clearance, at least in part, *via* stimulation of autophagic flux. Therefore, we examined p62 puncta in Purkinje neurons of WT mice and untreated and PG:PC-treated NPC1^I1061T/I1061T^ mice. The analysis revealed a significant (*p* < 0.01) reduction in p62 accumulation in calbindin-positive neurons of PC:PG-treated NPC1 mice compared with vehicle-treated controls (Fig. 6*B*), suggesting that the treatment enhances autophagic flux and ameliorates autophagic abnormalities. Consistent with the cellular models described above (Fig. S2*F*), immunofluorescence analysis of LAMP2 in samples from this animal model revealed a significant reduction of abnormal LY size and clustering after PG treatment (Fig. 6*B*). Thus, these data from a mouse model of NPC1 disease provide preclinical evidence that supports the therapeutic potential of PG/LBPA treatment to deplete neuronal sterol accumulation and re-establish lysosomal function in NPC1 disease.

## Discussion

We recently showed that cholesterol clearance in NPC1 cells following enrichment with LBPA is entirely dependent on its interaction with the hydrophobic knob domain of the NPC2 protein (25). LBPA enrichment, therefore, is apparently able to alleviate LE/LY cholesterol storage *via* an NPC2-dependent but NPC1-independent mechanism. It is not likely that LBPA is functioning through a proteostasis-based process, in which sufficient NPC1 protein is stabilized, evading proteosomal degradation and reaching its target organelle, since efficient cholesterol clearance occurs in NPC1 KO cells as well (Fig. S1*A* and (25, 27)). The mechanism underlying the PG/LBPA-mediated bypass of NPC1 protein, thought to be essential for cholesterol export from the LE/LY (6, 7, 62), is unknown.

**Figure 7:**
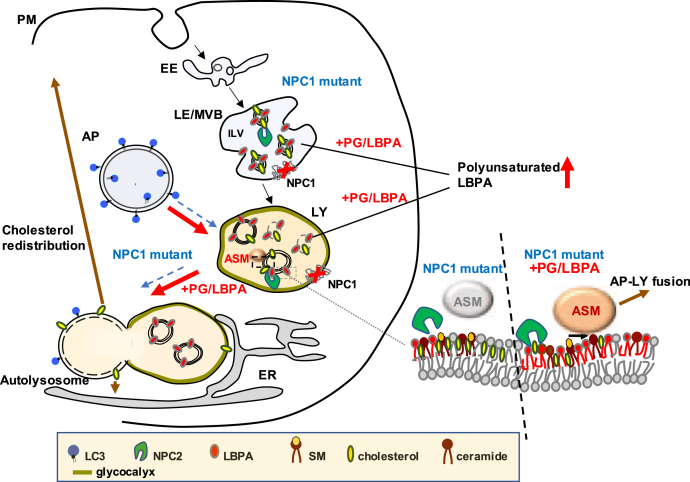
**Proposed mechanism of action of PG/LBPA enrichment in stimulating autophagy in NPC1 mutant cells**. Untreated NPC1-deficient (NPC mutant) cells with elevated cholesterol levels in the LE (MVB)/LY have impaired AP-LY fusion, reduced ASM activity, enlarged AP and LY, and insufficient LBPA. *Dashed blue arrows* indicate processes impaired in NPC1-deficient cells. In the presence of functional NPC2, PG/LBPA enrichment of NPC1 mutant cells leads to cholesterol clearance from the LE/LY. PG/LBPA enrichment activates ASM, which mediates enhancement of AP-LY fusion to form autolysosomes, and enhances autophagic flux (*red arrows*). Enhanced AP-LY fusion would provide an exit route for cholesterol out of the LE/LY that can more easily bypass the LY glycocalyx and access the membrane bilayer with consequent intracellular redistribution of cholesterol to the PM and/or to the ER and other organelles (*brown arrow*, proposed). AP, autophagosome; ASM, acid sphingomyelinase; ER, endoplasmic reticulum; LBPA, lysobisphosphatidic acid; LE, late endosome; LY, lysosome; MVB, multivesicular body; NPC1, Niemann–Pick type C1; PC, phosphatidylcholine; PG, phosphatidylglycerol; PM, plasma membrane.

Here we show that PG/LBPA enrichment induces formation of autolysosomes in NPC1 cellular models, providing at least a partial route for NPC1-independent cholesterol clearance and its intracellular redistribution to the PM and ER. Furthermore, we show that the increased autophagic maturation is mediated, at least in part, *via* stimulation of ASM activity. These findings are depicted graphically in Figure 7. Although we assume the actions are due to LBPA, we cannot yet exclude independent or perhaps additive effects of PG itself; the potential therapeutic benefit is evident, irrespective of its arising secondary to PG or LBPA. Future studies will address this question using nonhydrolyzable forms of PG to distinguish actions of the precursor PG from those of the product LBPA.

Based on the reported properties of LBPA as a membrane fusogenic lipid (18, 19, 41), we hypothesized that an increase in LBPA may stimulate AP-LY fusion and autophagic flux, known to be impaired in NPC1 disease (13, 14, 17). The results indicate that, indeed, LBPA enrichment in NPC1 cells significantly improved autophagic flux and restored lysosomal properties toward normal. The PG treatment does not impact autophagy initiation events, in keeping with studies showing that stimulation of mTORC1 with rapamycin increases autophagy but does not lead to cholesterol clearance in NPC1-deficient cells (14). The remarkable incorporation of polyunsaturated fatty acyl chains into LBPA, particularly DHA, generated following incubation with di-oleoyl PG points to the likelihood of a specific functional impact of the PUFA-incorporated species. Although no reports are available that directly assess the fusogenic properties of LBPA with different acyl chains, phospholipid membranes enriched in DHA have been shown to be predisposed to fusion (63) and studies by Antonny and coworkers demonstrate that the presence of highly unsaturated chains, particularly in combination with less saturated chains within the same phospholipid, leads to heightened membrane vesiculation and fusion (64, 65), both properties that play important roles in autophagic progression. As LBPA is reported to reside largely in the ILV membranes and also in the limiting membrane of the LE/MVB compartment (66), the remarkable enrichment of LBPA content, and the specific enrichment in PUFA-containing LBPA species in particular, could potentially alter the properties of these membranes so as to favor LE/LY fusion with other organelles, including AP.

NPC1 cells are known to have low levels of LY ASM activity despite its normal expression and localization (29, 30), and it has been shown that restoration of ASM activity could reverse cholesterol accumulation (29). Furthermore, low ASM activity has been associated with defects in maturation of autophagic membranes and defective autophagic flux in NPA disease, which is caused by a recessive mutation in the *SMPD1* gene encoding ASM (54, 55). ASM binds strongly to inner LY membranes by electrostatic forces and catalyzes the breakdown of sphingomyelin (SM) into ceramide and phosphocholine (50). The anionic phospholipids LBPA and PG have been shown to activate ASM in a liposomal assay system, whereas LY cholesterol overload contributes to inactivation of the enzyme (30, 50, 51). Thus, we reasoned that LBPA in inner endolysosomal membranes (18, 19, 20, 67) could function within the LE/LY compartment to stimulate ASM activity and counter the inhibitory effects of cholesterol accumulation. PG or LBPA liposomes were found to restore ASM activity in NPC1-deficient fibroblasts, and ASM inhibition abrogated the stimulatory effects of PG on both cholesterol clearance as well as autophagic flux. These results support the notion that cholesterol clearance and autophagic flux are related processes in NPC1 disease. Taken together these data suggest that ASM mediates, at least in part, the effects of PG/LBPA enrichment on cholesterol egress from the LE/LY, and that this is occurs *via* enhanced autophagic flux.

Work is in progress to understand how ASM regulates autophagic maturation in NPC1 cells. We are testing whether the transient receptor potential mucolipin-1 (TRPML1) channel, which mediates Ca^2+^ release from the LE/LY lumen into the cytosol and has recently been shown to positively regulate AP-LY fusion and autophagic flux (53, 68, 69), could contribute to this mechanism. It has been reported that the ASM substrate, SM, and ASM itself, modulate the LY TRPML1 channel and Ca^2+^ signaling in NPC1 and NPA/B diseases (70). Another potential mechanism is an ASM-induced increase in LY ceramide. Ceramide can alter membrane structure and curvature (71), such that ASM-mediated increases in LY ceramide facilitate LY fusion to the PM, endosomes, and phagosomes (72). Ceramide also interacts with LC3-II to mediate cargo recruitment to AP (73, 74). Thus, the ASM–ceramide pathway could potentially be mediating the effects of LBPA enrichment.

Enhanced AP-LY fusion would apparently provide a route for cholesterol transport out of the endolysosomal compartment that bypasses NPC1, which is thought to be required for cholesterol to penetrate through the glycocalyx-coated limiting LY membrane (6, 49). Cholesterol, in contrast to other lipids, cannot be degraded. However, the fusion of AP with LY, giving rise to the autolysosome, a hybrid organelle without a continuous glycocalyx, could promote the partition of cholesterol directly into the limiting membrane by luminal NPC2, which has been shown to be a membrane interactive protein (24, 75, 76). Consequently, cholesterol might become available for cholesterol transporters that localize to the limiting membrane or at membrane–membrane contact sites for further cholesterol transport to acceptor organelles such as ER, mitochondria, PM, or peroxisomes (11, 77, 78, 79). Such candidate transporters include Oxysterol-Binding Protein-Related Protein 1L (ORP1L), STARD3, and OSBP-related proteins 5 (ORP5) and 2 (ORP2) (11, 80, 81, 82, 83). A tight coupling between LY and other organelles has been described in many cells. In NPC1 fibroblasts the ER domains in direct contact with LY appeared significantly enlarged and more persistent than in control cells (79), although others have not found this to be the case (78, 82). Another study showed that LY-mitochondria membrane contact sites are also increased in NPC1 cells, and it has been suggested that expansion of LY-organelle interfaces might be a compensatory mechanism for the loss of NPC1 (78).

Autophagic maturation is not the sole route of endolysosomal cholesterol mobilization in PG/LBPA-enriched cells. LBPA has been shown to play a role in the biogenesis of multivesicular bodies (MVBs) (another term for LE), particularly in the formation of the ILVs (19, 27, 66, 84). As noted above, we recently found that enrichment of LBPA in NPC1-deficient cells induces MVB redistribution toward the PM with a consequent increase in exosome secretion, and, hence, increased discharge of cholesterol (28). Thus, exosomal secretion is an additional route of endolysosomal cholesterol efflux.

There are currently few treatment options for the devastating neurological effects of Niemann–Pick type C disease (85, 86). Direct delivery of cyclodextrins to the CNS has shown promise despite noted side effects (87, 88). Stabilization of the NPC1 protein using chaperone therapy is also being developed (89), however, even with ultimate success it will not treat patients with NPC1 who express none of the protein. The proof-of-concept experiment shown herein, where a single dose of PG-containing liposomes led to significant reduction in Purkinje neuron cholesterol levels in the NPC1^I1061T^ mouse, supports continued development of PG/LBPA enrichment as a potential therapeutic approach that could in principle be applied to all cases of NPC1 disease, and even to those cases of NPC2 disease where mutations lie outside the LBPA-interacting domain (25). The concomitant decrease in the AP marker p62 supports the involvement of improved autophagic clearance in the observed cholesterol reduction.

Collectively, our data indicate that PG/LBPA enrichment in three NPC1 cellular models results in re-establishment of lysosomal homeostasis as well as autophagic clearance of toxic protein aggregates, along with cholesterol. The findings suggest that PG/LBPA enrichment may also have great potential for the treatment of other lysosomal storage disorders as well as neurological diseases with autophagy defects, for example Parkinson’s or Alzheimer’s disease. NPC1 disease shares many similarities with Alzheimer’s disease (90), and increased endogenous LBPA has been reported in both NPC1 (27) and Alzheimer’s disease (91), where such an increase might be a compensatory homeostatic cellular response to eliminate toxic deposits.

Numerous studies demonstrate the importance of autophagy activation for treatment of neurodegenerative diseases (reviewed in (92)). The pharmacological strategies currently in use are mostly based on overall induction of the whole autophagy process, and excessive autophagy activation often results in toxic effects (92). PG treatment, by contrast, targets the culminating events of the autophagy process, and has thus far shown no toxicity in cellular and animal models.

These findings may also be informative for several viral infections where LBPA serves as a cofactor for virus entry and fusion with LE membranes including HIV, dengue, Uukuniemi, Lassa, and the novel SARS-Cov-2 (93, 94, 95, 96), in that PG liposomes can be used as a reagent that efficiently increases LBPA for studying virus–host interactions.

The identification of regulators of LBPA metabolism, currently not established (97), will be essential to develop strategies that allow for the manipulation of LBPA levels.

## Experimental procedures

### Antibodies and reagents

Rabbit polyclonal anti-SREBP2 (Abcam #ab30682), Rabbit monoclonal a-LC3A/B (D3U4C) (Cell Signaling Technology, #13173), Rabbit monoclonal anti-LC3B (D11) (Cell Signaling Technology, #3868), Mouse monoclonal anti-LC31A/1B (Cosmo Bio USA, #CAC-CTB-LC3-2-IC), Mouse monoclonal anti-SQSTM (Abnova, #H00008878-M01), Mouse monoclonal anti-SQSTM1 (D-3) (Santa Cruz Biotechnology, #sc-28359), Mouse monoclonal anti-SQSTM1/p62 (D5L7G) (Cell Signaling Technology, #88588), Rabbit polyclonal anti-NPC1 (Abcam, #ab36983), Rabbit polyclonal anti-Beclin1 (Novus, #NB110-87318), Rabbit monoclonal anti-LAMP1 (Sino Biological, #11215-R107), Mouse monoclonal anti-GAPDH (0411) (Santa Cruz Biotechnology, #sc-47724), Mouse monoclonal anti-β-Actin (Sigma-Aldrich, #A5441), Rabbit polyclonal anti-calbindin (Abcam, #ab49899), Rabbit polyclonal anti-calbindin-D-28K (EG-20) (Sigma-Aldrich, #C2724), Rabbit polyclonal anti-Phospho-AMPKα (Thr172) (Cell Signaling Technology, #2531), Mouse monoclonal anti-AMPKα (F6) (Cell Signaling Technology, #2793), Rat monoclonal anti-LAMP-2 (mouse) (DSHB, #ABL-93), Rabbit monoclonal anti-Phospho-p70 S6 Kinase (Thr389) (D5U1O) (Cell Signaling Technology, #97596), Rabbit monoclonal p70 S6 Kinase (49D7) (Cell Signaling Technology, #5707), Rabbit monoclonal Phospho-mTOR (Ser2448) (D9C2) (Cell Signaling Technology, #5536), Anti-Human SMPD1 Affinity Purified Polyclonal Antibody (R&D Systems, #AF5348), Mouse ATG5 (C-1) (Santa Cruz, sc-133158), Alexa Fluor secondary anti-rabbit, anti-mouse IgG (Invitrogen). Bafilomycin A1 (Cell Signaling Technology, #54645), Amitriptyline hydrochloride (Sigma, #A8404), EACC (Aobious, Cat#AOB13386), 18:1 (Δ9-*Cis*) 1,2-Dioleoyl-sn-Glycero-3-(Phospho-rac-(1-glycerol) (PG) (Avanti Polar Lipids, # 840475), L-a-Phosphatidylcholine (PC) (Avanti Polar Lipids, # 840051), 1 BMP (S,R) bis(monooleoylglycero)phosphate (S,R isomer) ammonium salt) (LBPA) (Avanti Polar Lipids, #857133C).

### Plasmids and lentiviral particles

mCherry-D4H was a gift from Gregory Fairn (University of Toronto), PH-PLCδ1-GFP was a gift from Tamas Balla (NIH), Human SMPD1 Gene ORF cDNA clone expression plasmid, C-Myc tag (Sino Biological), pCMV3-C-Myc Negative Control Vector (C-terminal Myc-tagged) (Sino Biological), scrambled control shRNA lentiviral particles (Santa Cruz Bio, sc-108080), Atg5 shRNA (Santa Cruz Bio, sc-41445-V).

### Mammalian cell culture

HeLa (ATTC #CCL-2) and HeLa NPC1 knockout cells, described previously (25), were cultured in Dulbecco's modified Eagle's medium (Gibco) supplemented with 10% (v/v) heat-inactivated FBS (Atlanta Biologicals) and 1% penicillin-streptomycin solution (Gibco). Human NPC1 WT (GM03652 and GM05659) and NPC1-deficient fibroblasts (GM03123) were obtained from Coriell Cell Repositories (Coriell Institute). Human fibroblasts were grown in MEM Eagle with Earle’s salts (Sigma) supplemented with 15% FBS, 1% penicillin–streptomycin, and 1X sodium pyruvate (Gibco). All cells were below passage 15. WT (GM05659) and NPC1 (GM03123) patient skin fibroblasts were differentiated to neural stem cells (NSC) *via* iPSC reprogramming using standard laboratory techniques (31, 98). NSCs were cultured in Matrigel (Corning)-coated T-75 flasks at passage 9 and seeded in 96-well PDL-coated plates (Greiner Bio One) using Knockout Dulbecco's modified Eagle's medium (Thermo Fisher Scientific) supplemented with bFGF (Thermo Fisher Scientific), EGF (Thermo Fisher Scientific), StemPro neural supplement (Thermo Fisher Scientific) and Glutamax plus (Thermo Fisher Scientific), 10 μg/ml vitronectin (Thermo Fisher Scientific), and 1:400 dilution of Matrigel at a density of 10,000 (WT) or 12,000 (NPC1) cells per well for experiments. All cell lines were maintained at 37 °C and 5% CO_2_. All cells were free of mycoplasma contamination, were cultured aseptically using only mycoplasma-free reagents, and were routinely checked using LookOut mycoplasma PCR detection kit (Sigma-Aldrich) and Hoechst staining.

### Mice

All Npc1-I1061T mice (59) were backcrossed to C57BL/6 (≥10 generations). All procedures involving mice were approved by the University of Michigan Committee on Use and Care of Animals.

### Cell viability quantification

A total of 8000 fibroblasts/well were seeded in white 96-well plates (Corning) and after 24 h were treated with PG or compounds. Cell viability was assessed by CellTiter-Glo luminescent cell viability assay (Promega) in accordance with the manufacturer’s instructions using FLUOstar Omega plate reader.

### Transfections

DNA transfections were performed with electroporation of 2 μg of DNA using Amaxa Human Dermal Fibroblast Nucleofector Kit (Lonza) and program U-023 for fibroblasts, or Nucleofector Kit V (Lonza) and program G-030 for HeLa cells using the Amaxa Nucleofector II/2b device (Lonza).

### Atg5 knockdown

NPC1-deficient fibroblasts or NPC1 KO HeLa cells were seeded in 12-well plates and after 24 h were transduced with Atg5 shRNA or scrambled control shRNA lentiviral particles (all from Santa Cruz Bio) in concentrations of 1 × 10^5^ infectious units of virus. Stable clones expressing the shRNA were selected *via* Puromycin dihydrochloride selection (2 μg/ml) according to the manufacturer’s protocol. Knockdown efficiency was validated by qPCR or Western blot analysis Atg5 antibody (Santa Cruz Bio).

### Preparation of liposomes and treatment

PG, PC, PC:PG, or PC:LBPA liposomes were prepared by transferring 18:1 (Δ9-*Cis*) 1,2-Dioleoyl-sn-Glycero-3-(Phospho-rac-(1-glycerol) (PG) (Avanti Polar Lipids), or L-a-Phosphatidylcholine (PC) (Avanti Polar Lipids), or a mixture of PC and PG (1:1 mol:mol), or a mixture of PC and 18:1 BMP (S,R) ammonium salt) (LBPA) (Avanti Polar Lipids) (1:1 mol:mol) in chloroform to a sterile glass vessel and evaporating the solvent using a stream of nitrogen gas for 40 min. The lipid film was then dispersed in sterile PBS to obtain a 5 mM concentration. After hydrating the lipid film for 1 h on ice and following vortexing for 5 min, the multivesicular liposomes were then sonicated for 40 min at 4 °C under nitrogen gas to make small unilamellar liposomes using Branson sonifier set at duty cycle 70 and output control 3. They were then centrifuged at 16,000*g* for 30 min to remove titanium particles and multilamellar liposomes. Cells were treated with liposomes when they reached 70% to 80% confluency. Prior to treatment, the medium was replaced with fresh medium. In experiments with HeLa and fibroblasts, liposomes were added to a concentration of 100 μM. In experiments with NSC 30 and 50 μM PG or PC-containing liposomes were used. Control untreated cells were treated with the same volume of PBS. For animal treatment the liposome suspension was additionally sterilized by passing it through the 0.45-μm pore-size filter (Millipore).

### Western blotting

Lysates were prepared from cell pellets using RIPA buffer supplemented with 1X protease and phosphatase inhibitor cocktail. Cell lysates were centrifuged at 16,000*g* for 12 min at 4 °C. Protein concentrations in supernatants were determined by BCA kit. Twenty micrograms of protein from each sample was separated by SDS-PAGE and electrophoretically transferred onto nitrocellulose membranes (Bio-Rad) and blocked with 5% (wt/vol) nonfat milk in Tris-buffered saline and 0.1% Tween-20 (TBST). Membranes were incubated with primary antibodies using 1:1000 dilution in TBST buffer containing 3% fatty acid–free BSA overnight at 4 °C with shaking, and with peroxidase-conjugated secondary antibodies (Invitrogen) using 1:10,000 dilution for 1 h at RT. The protein–antibody complexes were visualized using Western Bright Quantum kit (Advansta) and exposed to X-ray film. X-ray films were scanned using a transparency adapter feature, and protein densitometry was performed using NIH ImageJ software. Relative optical density was calculated by dividing the densitometry of protein with its respective loading control from the same blot. The values obtained were used to calculate the treatment *versus* control in percentage. The following LC3 and p62 antibodies, different from immunostaining experiments described below, were used for Western blot: rabbit monoclonal anti-LC3A/B (D3U4C) (Cell Signaling Technology, #13173) and mouse monoclonal anti-SQSTM1/p62 (D5L7G) (Cell Signaling Technology, #88588).

### Real-time RT-PCR analysis

Cellular RNA was isolated using a NucleoSpin RNA Plus kit (Macherey Nagel). cDNA synthesis was performed using High-Capacity cDNA Reverse Transcription Kit (Thermo Fisher Scientific) with 1 μg of total RNA. RT product was amplified using *SMPD1* Hs03679349_g1, *GAPDH* Hs02786624_g1, *POLR2A* Hs00172187_m1, and *ATG5* Hs00169468 TaqMan Gene Expression Assays (Thermo Fisher Scientific). Detection was performed using the StepOne Real-Time PCR System (Applied Biosystems). Differences in the levels of gene expression were determined by relative quantification using the delta Ct method.

### RFP-GFP-LC3 assay

To evaluate the number of autophagosomes and autolysosomes cells were treated with PG for 24 h and after 8 h infected with Premo Autophagy Tandem Sensor RFP-GFP-LC3 (Thermo Fisher Scientific) for 16 h. Live cells were examined under a Zeiss confocal laser scanning microscope (Zeiss, LSM 710) equipped with a 63X oil immersion objective.

### Flow cytometry

Cells were incubated with 75 nM LysoTracker Red DND-99 (Invitrogen) for 30 min at 37 °C under regular cell culture conditions. Then, cells were washed 3x in PBS, detached with trypsin, washed and resuspended in PBS, and analyzed using Accuri C6 flow cytometer.

### Filipin and PFO∗ staining

Cells were fixed with 4% paraformaldehyde (Electron Microscopy Sciences) in PBS for 30 min. For filipin staining cells on coverslips were stained with 25 μg/ml filipin (Sigma) in PBS in the dark for 2 h at RT, washed with PBS, mounted in Fluoromount G (EMS), and imaged. In some experiments nuclear staining was performed using SYTOX Green or 7-AAD from SelectFX Nuclear Labeling Kit (Invitrogen). For PFO∗ staining cells were permeabilized with 0.1% TX-100 in Cell Staining Buffer (BioLegend) for 30 min and then blocked for 1 h in Cell Staining Buffer. iFluor-647-PFO∗ (Codex Bio solutions and AAT Bioquest, custom order) was then added in Cell Staining Buffer for 1 h. Cells were washed three times with PBS and stained with 1:10,000 HCS CellMask Green (ThermoFisher) and 1:5000 Hoechst 33342 (ThermoFisher) in PBS for 15 min. Cells were washed with PBS and imaged.

### Immunofluorescence

Fibroblasts and HeLa cells grown on coverslips were fixed with 4% PFA solution in 1x PBS for 10 min at RT and then washed three times with PBS. For LAMP1 (Sino Biological), LC3 (Cosmo Bio, #CAC-CTB-LC3-2-IC), and p62 (Abnova, #H00008878-M01) staining, primary antibodies were diluted in incubation buffer (0.2% Saponin [Sigma-Aldrich], 1% BSA in PBS) and incubated for 2 h at RT in dilutions of 1:300, 1:200, and 1:150, respectively. Cells were then rinsed three times with PBS. Fluorophore-tagged secondary antibodies AlexaFluor-488/555 (Invitrogen) were diluted, incubated, and rinsed the same way as with the primary antibodies. When needed, samples were incubated with designated stains before mounting the samples. Samples were mounted on glass slides using Fluoromount-G (Electron Microscopy Sciences). For p62 and LC3 staining in NSCs cells growing in 96-well plates, cells were fixed with 100% ice-cold methanol for 15 min at −20 °C, washed three times with PBS, blocked 30 min at RT with Cell Staining Buffer (Biolegend) and then stained with 1:250 mouse-anti-SQSTM1 antibody (anti-SQSTM1 (D-3), Santa Cruz Biotechnology, #sc-28359) and 1:500 rabbit-anti-LC3B antibody (Cell Signaling Technology, #13173) in Cell Staining Buffer for 2 h. Cells were washed in PBS three times and stained with secondary 1:1000 AlexaFluor-488 (Invitrogen), goat-anti-rabbit and 647 goat-anti-mouse (Invitrogen) in Cell Staining buffer for 1 h. Hoechst 33342, 1:5000 in PBS, was added for 15 min, and plates were sealed for imaging.

### Fluorescence imaging and analysis

Filipin imaging in cells was performed on an Echo Revolve Hybrid upright/inverted microscope containing four Epi-Fluorescence lines. Confocal imaging was performed on a Zeiss LSM710, Zeiss Cell Observer SD confocal system, or LSM810 Confocal Laser Scanning microscope (Carl Zeiss) equipped with deep UV and 458-, 488-, 514-, 565-, and 633-nm laser lines. Cells for fixed samples were grown on glass coverslips, processed according to experimental requirements as described above, and mounted on glass slides. When comparing different treatments, images were captured using identical microscope settings. Images were analyzed with ImageJ (NIH) or NIS Elements (Nikon) software. To quantify the relative fluorescence of the target structures for filipin staining, regions of interest (ROIs) were selected for the target areas (ROIt) and for background areas (ROIb). Target areas were puncta of filipin signal originated from the LE/LY area. The nuclear area was excluded during quantification. Results are corrected for background fluorescence. At least 80 cells were analyzed per condition in an average number of 20 images/fields and data were analyzed from 2 to 4 separate experiments. LC3 and p62 puncta were quantified as average/cell manually or using the particle analysis feature of ImageJ software.

### Automated imaging and image analysis in NSCs

Twenty-four hours before PG or PC treatment NSCs plated in 96-well plates were incubated in the presence of 2.5% FBS to induce cholesterol accumulation. After PG or PC addition cells were incubated for an additional 24 or 48 h. Plates were processed using standard immunocytochemistry methods for cholesterol and p62/LC3B immunostaining. NSCs were stained in 96-well plates, and plates were imaged on the IN Cell 2500HS (GE Healthcare) using a 20x air objective. Filter sets included the DAPI, green, and far-red filters. Six fields per well were captured using 1 × 1 binning. Laser and Software Autofocus was used to focus on the cells. 2D deconvolution was also used during image acquisition to focus on the puncta and resolve the small structures in both PFO∗ and p62/LC3B staining. Images were loaded onto the Columbus Analyzer software for quantification of cell number, cell morphology, and staining intensity. Nuclei were automatically segmented using the blue channel to begin object identification. Cell cytoplasm was segmented using the green channel for PFO∗-stained cells and the far-red channel for the p62/LC3B-stained cells. Further segmentation was performed for the green and far-red channels to identify the spots. Morphology and intensity parameters were quantified for each object and channel. Data were exported as a .csv into Microsoft Excel, and graphs were generated using GraphPad Prism 8. For representative images fluorescent colors of p62 and LC3B were changed.

### Acid sphingomyelinase activity assay

The enzymatic hydrolysis of SM to ceramide and phosphocholine by acid sphingomyelinase was measured with the Acid Sphingomyelinase Activity Assay Kit (Echelon Biosciences, K-3200) according to manufacturer’s instructions. HeLa and fibroblast cells growing in 6-well plates were washed with cold PBS and then scraped in 1 mM PMSF on ice. Subsequently, cells were sonicated in an ice water bath Branson sonicator for 10 min and then were disrupted by three rounds of freeze–thaw cycles with liquid nitrogen with vortexing in between each cycle. Extracts were clarified by centrifugation for 12 min at 16,000*g* at 4 °C, and protein concentrations were determined by the BCA protein assay (Thermo Scientific). The enzymatic assay was carried out in 96-well plates. Each well contained 5 μg of protein in 20 μl of 1 mM PMSF. ASM standards were prepared in1 mM PMSF as well. Substrate buffer (K-3203), 30 μl, was added to each well containing 20 μl of sample or standard. Then 50 μl/well of the diluted substrate (K-3202) in substrate buffer was added to each well. The plate was incubated for 3 h at 37 °C with agitation. Stop buffer (K-3204) was added to each well, and fluorescence was measured after 10 min of shaking at RT using FLUOstar Omega plate reader at 360 nm excitation and 460 emission. Data were represented as pmol substrate degraded/μg protein/h.

### Acid ceramidase activity assay

AC activity was determined as described (99). Cells were trypsinized and washed twice with PBS. Cell pellets were resuspended in 100 μl of 0.2 M sucrose solution, sonicated in an ice water bath Branson sonicator for 10 min, and then disrupted by three rounds of freeze–thaw cycles with liquid nitrogen with vortexing in between each cycle. Cell homogenates were centrifuged at 16,000*g* for 3 min. Protein was determined in the supernatant by the BCA assay. Enzymatic assay was carried out in black 96-well plates. Each well contained a mixture of 74.5 μl of 25 mM sodium acetate buffer pH 4.5, 0.5 μl of a 4 mM Rbm14-12 substrate (provided by Antonio Delgado, University of Barcelona) solution in ethanol (substrate final concentration 20 μM; ethanol final concentration 0.5%), and 10 μg of protein in a volume of 25 μl of 0.2 M sucrose solution. The plate was incubated at 37 °C for 3 h without agitation. Then, the enzymatic reaction was stopped by adding 50 μl of methanol and 100 μl of a 2.5 mg/ml NaIO_4_ fresh solution in 100 mM glycine/NaOH buffer pH 10.6 in each well. The plate was incubated for 2 h in dark, and then the fluorescence was quantified using a FLUOstar Omega plate reader at 360 nm excitation and 446 nm emission. The amount of umbelliferone released was calculated from the fluorescence intensity by using calibration curves with umbelliferone (Sigma) standard. For the calibration curve, 10 mM umbelliferone solution was prepared in ethanol. Serial dilutions were performed in 25 mM sodium acetate buffer pH 4.5 in the range of 0 to 3000 pmol. To each well containing 100 μl of umbelliferone standards 50 μl of methanol and 100 μl of 100 mM glycine/NaOH buffer pH 10.6 were added.

### Cardiolipin analysis

CL analysis was performed as described (100). Briefly, a small aliquot of the lipid extract was mixed 1:1 with matrix solution containing 20 g/l 9-aminoacridine in 2-propanol/acetonitrile (3:2, v/v). For each sample, 1 μl of this mixture was spotted on a target plate. Measurements were performed with a MALDI Micro MX mass spectrometer (Waters) operated in reflectron mode. The pulse voltage was set to 2000 V, the detector voltage was set to 2200 V, and the TLF delay was set to 700 ns. The nitrogen laser (337 nm) was fired at a rate of 5 Hz, and ten laser shots were acquired per subspectrum. The instrument was operated in negative ion mode with a flight tube voltage of 12 kV, a reflectron voltage of 5.2 kV, and a negative anode voltage of 3.5 kV and calibrated daily. We typically acquired 100 subspectra (representing 1000 laser shots) per sample in a mass range from 400 to 2000 Da. Spectra were only acquired if their base peak intensity was within 10% to 95% of the saturation level. Data were analyzed with the MassLynx 4.1 software.

### Phospholipid analysis

Lipidomics profiling was performed using ultra performance liquid chromatography–tandem mass spectrometry (UPLC-MSMS). Lipid extracts were prepared from cell lysates using a modified Bligh and Dyer method (101), spiked with appropriate internal standards, and analyzed on a platform comprising Agilent 1260 Infinity HPLC integrated to Agilent 6490A QQQ mass spectrometer controlled by Masshunter v 7.0 (Agilent Technologies). Quantification of lipid species was accomplished using multiple reaction monitoring transitions (91, 102, 103) under both positive and negative ionization modes in conjunction with referencing of appropriate internal standards: PA 14:0/14:0, PC 14:0/14:0, PE 14:0/14:0, PG 15:0/15:0, PI 17:0/20:4, PS 14:0/14:0, LBPA 14:0/14:0, AcylPG 14:0/14:0, LPC 17:0, LPE 14:0, LPI 13:0 (Avanti Polar Lipids). Lipid levels for each sample were calculated by summing up the total number of moles of all lipid species measured by all three LC-MS methodologies and then normalizing that total to mol %. The final data are presented as mean mol % with error bars showing mean ± SD.

### Stereotaxic mouse intracerebral ventricular bolus delivery

Sterile PC:PG liposomes in PBS (pH 7.4) (1:1 mol:mol, 400 μM PC: 400 μM PG) were stereotaxically administered to the right lateral ventricle *via* intracerebral ventricular injection on mice under vaporized isoflurane anesthesia according to Institutional Animal Care and Use Committees guidelines. Using established protocols, 7-week-old mice received a single intracerebral ventricular bolus injection of either PC:PG or PBS vehicle (104). Briefly, the anesthetized mouse received a small scalp incision to reveal Bragma sutures and a small burr hole was drilled based on the following coordinates relative to Bragma: anteroposterior +0.3 mm, mediolateral −1.0 mm. To reduce tissue shearing upon needle placement, a point style 4, 45-degree beveled needle (Hamilton Lab Products, #7758-04) connected to a 10-μl syringe (Hamilton) was placed dorsoventral −3.0 mm at a rate of 1 mm/s. To prevent backflow of treatment around the injection site, the brain tissue was allowed 3 min to seal around the needle. Using an injection pump (UMC4, World Precision Instruments, Inc), 10 μl of vehicle or PC:PG liposomes was delivered at an infusion rate of 0.5 μl/s. The needle was retracted 5 min after delivery at a rate of 1 mm/s, and the incision site was sutured with synthetic nonabsorbable sutures (Henry Schein). According to Institutional Animal Care and Use Committees guidelines, mice were recovered in a temperature-controlled environment and following surgery, the mouse weight, grooming activity, and home cage activity were recorded for up to 7 days. No postsurgical adverse events occurred in any of the treated mice.

### Filipin staining and immunofluorescence in mouse brain tissue

One week after intraventricular injection with vehicle or PC:PG, mice were perfused with saline and their brains were placed in 4% paraformaldehyde overnight. The right hemisphere of the brain was frozen in optimal cutting temperature formulation, cut into 10-μm-thick sagittal sections, and captured onto microscope slides. Sections were immersed in permeabilization buffer (0.1% Triton/10% normal goat serum/1% BSA in PBS) for 10 min, then placed in blocking buffer (10% normal goat serum/1% BSA in PBS) for 50 min. Primary antibody (calbindin, Sigma-Aldrich, #C2724) was added to the sections, which were then incubated overnight at 4 °C. Following this incubation, sections were washed in PBS three times for 5 min, then immersed in secondary antibody for 1 h at RT. Sections were again washed in PBS and stained with filipin for 2 h in the dark at RT. Following another round of PBS washes, slides were mounted using ProLong Gold Antifade mounting medium (Invitrogen) and imaged on a Nikon A-1 confocal microscope. Using ImageJ in a blinded fashion, cells and their nuclei were outlined in the calbindin channel and raw intensity and area were measured in the filipin channel using the MultiMeasure plugin. Representative background was selected from the filipin channel and measured using the MultiMeasure plugin. Relative intensity was calculated with the following equation:

$$Relative\;intensity=\frac{\left(\frac{Raw\;intensity_{cell}}{Area_{cell}}\right)-\left(\frac{Raw\;intensity_{nucleus}}{Area_{nucleus}}\right)}{\left(\frac{Raw\;intensity_{background}}{Area_{background}}\right)}$$

For p62/LAMP2/filipin/calbindin costaining mouse monoclonal anti-SQSTM1 (Abnova, #H00008878-M01), rabbit polyclonal anti-calbindin (Abcam, #C2624), rat monoclonal anti-LAMP2 (DSHB), and filipin (Sigma-Aldrich) were used. Images were taken at Zeiss Cell Observer SD confocal system and analyzed as above.

### Statistical analyses

Data were analyzed using a two-tailed Student's *t* test or one-way ANOVA followed by Tukey’s multiple comparisons test where indicated using GraphPad Prism 8 Software. Results with a *p* value <0.05 were assigned statistical significance.

## Data availability

All of the data are contained within the article.

## Supporting information

This article contains supporting information.

## Conflict of interest

The authors declare that they have no conflicts of interest with the contents of this article.

## References

[1] Carstea E.D., Morris J.A., Coleman K.G., Loftus S.K., Zhang D., Cummings C., Gu J., Rosenfeld M.A., Pavan W.J., Krizman D.B., Nagle J., Polymeropoulos M.H., Sturley S.L., Ioannou Y.A., Higgins M.E. (1997). Niemann-Pick C1 disease gene: Homology to mediators of cholesterol homeostasis. Science.

[2] Naureckiene S., Sleat D.E., Lackland H., Fensom A., Vanier M.T., Wattiaux R., Jadot M., Lobel P. (2000). Identification of HE1 as the second gene of Niemann-Pick C disease. Science.

[3] Vanier M.T. (2015). Complex lipid trafficking in Niemann-Pick disease type C. J. Inherit. Metab. Dis..

[4] Pfrieger F.W., Vitale N. (2018). Cholesterol and the journey of extracellular vesicles. J. Lipid Res..

[5] Mobius W., van Donselaar E., Ohno-Iwashita Y., Shimada Y., Heijnen H.F., Slot J.W., Geuze H.J. (2003). Recycling compartments and the internal vesicles of multivesicular bodies harbor most of the cholesterol found in the endocytic pathway. Traffic.

[6] Kwon H.J., Abi-Mosleh L., Wang M.L., Deisenhofer J., Goldstein J.L., Brown M.S., Infante R.E. (2009). Structure of N-terminal domain of NPC1 reveals distinct subdomains for binding and transfer of cholesterol. Cell.

[7] Infante R.E., Wang M.L., Radhakrishnan A., Kwon H.J., Brown M.S., Goldstein J.L. (2008). NPC2 facilitates bidirectional transfer of cholesterol between NPC1 and lipid bilayers, a step in cholesterol egress from lysosomes. Proc. Natl. Acad. Sci. U. S. A..

[8] Wang M.L., Motamed M., Infante R.E., Abi-Mosleh L., Kwon H.J., Brown M.S., Goldstein J.L. (2010). Identification of surface residues on Niemann-Pick C2 essential for hydrophobic handoff of cholesterol to NPC1 in lysosomes. Cell Metab..

[9] Li X., Wang J., Coutavas E., Shi H., Hao Q., Blobel G. (2016). Structure of human Niemann-Pick C1 protein. Proc. Natl. Acad. Sci. U. S. A..

[10] Ikonen E. (2008). Cellular cholesterol trafficking and compartmentalization. Nat. Rev. Mol. Cell Biol..

[11] Pfisterer S.G., Peranen J., Ikonen E. (2016). LDL-cholesterol transport to the endoplasmic reticulum: Current concepts. Curr. Opin. Lipidol..

[12] Walkley S.U., Vanier M.T. (2009). Secondary lipid accumulation in lysosomal disease. Biochim. Biophys. Acta.

[13] Dai S., Dulcey A.E., Hu X., Wassif C.A., Porter F.D., Austin C.P., Ory D.S., Marugan J., Zheng W. (2017). Methyl-beta-cyclodextrin restores impaired autophagy flux in Niemann-Pick C1-deficient cells through activation of AMPK. Autophagy.

[14] Sarkar S., Carroll B., Buganim Y., Maetzel D., Ng A.H., Cassady J.P., Cohen M.A., Chakraborty S., Wang H., Spooner E., Ploegh H., Gsponer J., Korolchuk V.I., Jaenisch R. (2013). Impaired autophagy in the lipid-storage disorder Niemann-Pick type C1 disease. Cell Rep..

[15] Li J., Pfeffer S.R. (2016). Lysosomal membrane glycoproteins bind cholesterol and contribute to lysosomal cholesterol export. Elife.

[16] Maetzel D., Sarkar S., Wang H., Abi-Mosleh L., Xu P., Cheng A.W., Gao Q., Mitalipova M., Jaenisch R. (2014). Genetic and chemical correction of cholesterol accumulation and impaired autophagy in hepatic and neural cells derived from Niemann-Pick type C patient-specific iPS cells. Stem Cell Rep..

[17] Lee H., Lee J.K., Park M.H., Hong Y.R., Marti H.H., Kim H., Okada Y., Otsu M., Seo E.J., Park J.H., Bae J.H., Okino N., He X., Schuchman E.H., Bae J.S. (2014). Pathological roles of the VEGF/SphK pathway in Niemann-Pick type C neurons. Nat. Commun..

[18] Kobayashi T., Stang E., Fang K.S., de Moerloose P., Parton R.G., Gruenberg J. (1998). A lipid associated with the antiphospholipid syndrome regulates endosome structure and function. Nature.

[19] Le Blanc I., Luyet P.P., Pons V., Ferguson C., Emans N., Petiot A., Mayran N., Demaurex N., Faure J., Sadoul R., Parton R.G., Gruenberg J. (2005). Endosome-to-cytosol transport of viral nucleocapsids. Nat. Cell Biol..

[20] Kobayashi T., Beuchat M.H., Chevallier J., Makino A., Mayran N., Escola J.M., Lebrand C., Cosson P., Kobayashi T., Gruenberg J. (2002). Separation and characterization of late endosomal membrane domains. J. Biol. Chem..

[21] Brotherus J., Renkonen O. (1977). Subcellular distributions of lipids in cultured BHK cells: Evidence for the enrichment of lysobisphosphatidic acid and neutral lipids in lysosomes. J. Lipid Res..

[22] Cheruku S.R., Xu Z., Dutia R., Lobel P., Storch J. (2006). Mechanism of cholesterol transfer from the Niemann-Pick type C2 protein to model membranes supports a role in lysosomal cholesterol transport. J. Biol. Chem..

[23] Xu Z., Farver W., Kodukula S., Storch J. (2008). Regulation of sterol transport between membranes and NPC2. Biochemistry.

[24] McCauliff L.A., Xu Z., Li R., Kodukula S., Ko D.C., Scott M.P., Kahn P.C., Storch J. (2015). Multiple surface regions on the Niemann-Pick C2 protein facilitate intracellular cholesterol transport. J. Biol. Chem..

[25] McCauliff L.A., Langan A., Li R., Ilnytska O., Bose D., Waghalter M., Lai K., Kahn P.C., Storch J. (2019). Intracellular cholesterol trafficking is dependent upon NPC2 interaction with lysobisphosphatidic acid. Elife.

[26] Thornburg T., Miller C., Thuren T., King L., Waite M. (1991). Glycerol reorientation during the conversion of phosphatidylglycerol to bis(monoacylglycerol)phosphate in macrophage-like RAW 264.7 cells. J. Biol. Chem..

[27] Chevallier J., Chamoun Z., Jiang G., Prestwich G., Sakai N., Matile S., Parton R.G., Gruenberg J. (2008). Lysobisphosphatidic acid controls endosomal cholesterol levels. J. Biol. Chem..

[28] Ilnytska O., Jeziorek M., Lai K., Altan-Bonnet N., Dobrowolski R., Storch J. (2021). Lysobisphosphatidic acid (LBPA) enrichment promotes cholesterol egress via exosomes in Niemann Pick type C1 deficient cells. Biochim. Biophys. Acta Mol. Cell Biol. Lipids.

[29] Devlin C., Pipalia N.H., Liao X., Schuchman E.H., Maxfield F.R., Tabas I. (2010). Improvement in lipid and protein trafficking in Niemann-Pick C1 cells by correction of a secondary enzyme defect. Traffic.

[30] Reagan J.W., Hubbert M.L., Shelness G.S. (2000). Posttranslational regulation of acid sphingomyelinase in niemann-pick type C1 fibroblasts and free cholesterol-enriched Chinese hamster ovary cells. J. Biol. Chem..

[31] Yu D., Swaroop M., Wang M., Baxa U., Yang R., Yan Y., Coksaygan T., DeTolla L., Marugan J.J., Austin C.P., McKew J.C., Gong D.W., Zheng W. (2014). Niemann-Pick disease type C: Induced pluripotent stem cell-derived neuronal cells for modeling neural disease and evaluating drug efficacy. J. Biomol. Screen..

[32] Li J., Lee P.L., Pfeffer S.R. (2017). Quantitative measurement of cholesterol in cell populations using flow cytometry and fluorescent perfringolysin O. Methods Mol. Biol..

[33] Piazza G.J., Marmer W.N. (2007). Conversion of phosphatidylcholine to phosphatidylglycerol with phospholipase D and glycerol. J. Am. Oil Chem. Soc..

[34] Stanacev N.Z., Chang Y.Y., Kennedy E.P. (1967). Biosynthesis of cardiolipin in Escherichia coli. J. Biol. Chem..

[35] Schlame M., Greenberg M.L. (2017). Biosynthesis, remodeling and turnover of mitochondrial cardiolipin. Biochim. Biophys. Acta Mol. Cell Biol. Lipids.

[36] Eberle D., Hegarty B., Bossard P., Ferre P., Foufelle F. (2004). SREBP transcription factors: Master regulators of lipid homeostasis. Biochimie.

[37] Infante R.E., Radhakrishnan A. (2017). Continuous transport of a small fraction of plasma membrane cholesterol to endoplasmic reticulum regulates total cellular cholesterol. Elife.

[38] Maekawa M., Fairn G.D. (2015). Complementary probes reveal that phosphatidylserine is required for the proper transbilayer distribution of cholesterol. J. Cell Sci..

[39] Wojtanik K.M., Liscum L. (2003). The transport of low density lipoprotein-derived cholesterol to the plasma membrane is defective in NPC1 cells. J. Biol. Chem..

[40] Várnai P., Lin X., Lee S.B., Tuymetova G., Bondeva T., Spät A., Rhee S.G., Hajnóczky G., Balla T. (2002). Inositol lipid binding and membrane localization of isolated pleckstrin homology (PH) domains. Studies on the PH domains of phospholipase C delta 1 and p130. J. Biol. Chem..

[41] Falguieres T., Luyet P.P., Gruenberg J. (2009). Molecular assemblies and membrane domains in multivesicular endosome dynamics. Exp. Cell Res..

[42] Elrick M.J., Yu T., Chung C., Lieberman A.P. (2012). Impaired proteolysis underlies autophagic dysfunction in Niemann-Pick type C disease. Hum. Mol. Genet..

[43] Klionsky D.J., Elazar Z., Seglen P.O., Rubinsztein D.C. (2008). Does bafilomycin A1 block the fusion of autophagosomes with lysosomes?. Autophagy.

[44] Kimura S., Noda T., Yoshimori T. (2007). Dissection of the autophagosome maturation process by a novel reporter protein, tandem fluorescent-tagged LC3. Autophagy.

[45] Sanman L.E., van der Linden W.A., Verdoes M., Bogyo M. (2016). Bifunctional probes of cathepsin protease activity and pH reveal alterations in endolysosomal pH during bacterial infection. Cell Chem. Biol..

[46] Wheeler S., Haberkant P., Bhardwaj M., Tongue P., Ferraz M.J., Halter D., Sprong H., Schmid R., Aerts J., Sullo N., Sillence D.J. (2019). Cytosolic glucosylceramide regulates endolysosomal function in Niemann-Pick type C disease. Neurobiol. Dis..

[47] Vats S., Manjithaya R. (2019). A reversible autophagy inhibitor blocks autophagosome-lysosome fusion by preventing Stx17 loading onto autophagosomes. Mol. Biol. Cell.

[48] Mizushima N., Noda T., Yoshimori T., Tanaka Y., Ishii T., George M.D., Klionsky D.J., Ohsumi M., Ohsumi Y. (1998). A protein conjugation system essential for autophagy. Nature.

[49] Li X., Saha P., Li J., Blobel G., Pfeffer S.R. (2016). Clues to the mechanism of cholesterol transfer from the structure of NPC1 middle lumenal domain bound to NPC2. Proc. Natl. Acad. Sci. U. S. A..

[50] Linke T., Wilkening G., Lansmann S., Moczall H., Bartelsen O., Weisgerber J., Sandhoff K. (2001). Stimulation of acid sphingomyelinase activity by lysosomal lipids and sphingolipid activator proteins. Biol. Chem..

[51] Oninla V.O., Breiden B., Babalola J.O., Sandhoff K. (2014). Acid sphingomyelinase activity is regulated by membrane lipids and facilitates cholesterol transfer by NPC2. J. Lipid Res..

[52] Li X., Xu M., Pitzer A.L., Xia M., Boini K.M., Li P.L., Zhang Y. (2014). Control of autophagy maturation by acid sphingomyelinase in mouse coronary arterial smooth muscle cells: Protective role in atherosclerosis. J. Mol. Med. (Berl.).

[53] Xu M., Zhang Q., Li P.L., Nguyen T., Li X., Zhang Y. (2016). Regulation of dynein-mediated autophagosomes trafficking by ASM in CASMCs. Front. Biosci. (Landmark Ed.).

[54] Gabande-Rodriguez E., Boya P., Labrador V., Dotti C.G., Ledesma M.D. (2014). High sphingomyelin levels induce lysosomal damage and autophagy dysfunction in Niemann Pick disease type A. Cell Death Differ..

[55] Corcelle-Termeau E., Vindelov S.D., Hamalisto S., Mograbi B., Keldsbo A., Brasen J.H., Favaro E., Adam D., Szyniarowski P., Hofman P., Krautwald S., Farkas T., Petersen N.H., Rohde M., Linkermann A. (2016). Excess sphingomyelin disturbs ATG9A trafficking and autophagosome closure. Autophagy.

[56] Jenkins R.W., Idkowiak-Baldys J., Simbari F., Canals D., Roddy P., Riner C.D., Clarke C.J., Hannun Y.A. (2011). A novel mechanism of lysosomal acid sphingomyelinase maturation: Requirement for carboxyl-terminal proteolytic processing. J. Biol. Chem..

[57] Riethmuller J., Anthonysamy J., Serra E., Schwab M., Doring G., Gulbins E. (2009). Therapeutic efficacy and safety of amitriptyline in patients with cystic fibrosis. Cell. Physiol. Biochem..

[58] Ordonez Y.F., Abad J.L., Aseeri M., Casas J., Garcia V., Casasampere M., Schuchman E.H., Levade T., Delgado A., Triola G., Fabrias G. (2019). Activity-based imaging of acid ceramidase in living cells. J. Am. Chem. Soc..

[59] Praggastis M., Tortelli B., Zhang J., Fujiwara H., Sidhu R., Chacko A., Chen Z., Chung C., Lieberman A.P., Sikora J., Davidson C., Walkley S.U., Pipalia N.H., Maxfield F.R., Schaffer J.E. (2015). A murine Niemann-Pick C1 I1061T knock-in model recapitulates the pathological features of the most prevalent human disease allele. J. Neurosci..

[60] Ko D.C., Milenkovic L., Beier S.M., Manuel H., Buchanan J., Scott M.P. (2005). Cell-autonomous death of cerebellar purkinje neurons with autophagy in Niemann-Pick type C disease. PLoS Genet..

[61] Pacheco C.D., Kunkel R., Lieberman A.P. (2007). Autophagy in Niemann-Pick C disease is dependent upon beclin-1 and responsive to lipid trafficking defects. Hum. Mol. Genet..

[62] Pfeffer S.R. (2019). NPC intracellular cholesterol transporter 1 (NPC1)-mediated cholesterol export from lysosomes. J. Biol. Chem..

[63] Stillwell W., Wassall S.R. (2003). Docosahexaenoic acid: Membrane properties of a unique fatty acid. Chem. Phys. Lipids.

[64] Manni M.M., Tiberti M.L., Pagnotta S., Barelli H., Gautier R., Antonny B. (2018). Acyl chain asymmetry and polyunsaturation of brain phospholipids facilitate membrane vesiculation without leakage. Elife.

[65] Pinot M., Vanni S., Pagnotta S., Lacas-Gervais S., Payet L.A., Ferreira T., Gautier R., Goud B., Antonny B., Barelli H. (2014). Lipid cell biology. Polyunsaturated phospholipids facilitate membrane deformation and fission by endocytic proteins. Science.

[66] Bissig C., Lenoir M., Velluz M.C., Kufareva I., Abagyan R., Overduin M., Gruenberg J. (2013). Viral infection controlled by a calcium-dependent lipid-binding module in ALIX. Dev. Cell.

[67] Kobayashi T., Beuchat M.H., Lindsay M., Frias S., Palmiter R.D., Sakuraba H., Parton R.G., Gruenberg J. (1999). Late endosomal membranes rich in lysobisphosphatidic acid regulate cholesterol transport. Nat. Cell Biol..

[68] Scotto Rosato A., Montefusco S., Soldati C., Di Paola S., Capuozzo A., Monfregola J., Polishchuk E., Amabile A., Grimm C., Lombardo A., De Matteis M.A., Ballabio A., Medina D.L. (2019). TRPML1 links lysosomal calcium to autophagosome biogenesis through the activation of the CaMKKbeta/VPS34 pathway. Nat. Commun..

[69] Zhang X., Chen W., Gao Q., Yang J., Yan X., Zhao H., Su L., Yang M., Gao C., Yao Y., Inoki K., Li D., Shao R., Wang S., Sahoo N. (2019). Rapamycin directly activates lysosomal mucolipin TRP channels independent of mTOR. PLoS Biol..

[70] Shen D., Wang X., Li X., Zhang X., Yao Z., Dibble S., Dong X.P., Yu T., Lieberman A.P., Showalter H.D., Xu H. (2012). Lipid storage disorders block lysosomal trafficking by inhibiting a TRP channel and lysosomal calcium release. Nat. Commun..

[71] Goni F.M., Alonso A. (2002). Sphingomyelinases: Enzymology and membrane activity. FEBS Lett..

[72] Utermohlen O., Herz J., Schramm M., Kronke M. (2008). Fusogenicity of membranes: The impact of acid sphingomyelinase on innate immune responses. Immunobiology.

[73] Sentelle R.D., Senkal C.E., Jiang W., Ponnusamy S., Gencer S., Selvam S.P., Ramshesh V.K., Peterson Y.K., Lemasters J.J., Szulc Z.M., Bielawski J., Ogretmen B. (2012). Ceramide targets autophagosomes to mitochondria and induces lethal mitophagy. Nat. Chem. Biol..

[74] Dany M., Gencer S., Nganga R., Thomas R.J., Oleinik N., Baron K.D., Szulc Z.M., Ruvolo P., Kornblau S., Andreeff M., Ogretmen B. (2016). Targeting FLT3-ITD signaling mediates ceramide-dependent mitophagy and attenuates drug resistance in AML. Blood.

[75] Abdul-Hammed M., Breiden B., Adebayo M.A., Babalola J.O., Schwarzmann G., Sandhoff K. (2010). Role of endosomal membrane lipids and NPC2 in cholesterol transfer and membrane fusion. J. Lipid Res..

[76] McCauliff L.A., Xu Z., Storch J. (2011). Sterol transfer between cyclodextrin and membranes: Similar but not identical mechanism to NPC2-mediated cholesterol transfer. Biochemistry.

[77] Chu B.B., Liao Y.C., Qi W., Xie C., Du X., Wang J., Yang H., Miao H.H., Li B.L., Song B.L. (2015). Cholesterol transport through lysosome-peroxisome membrane contacts. Cell.

[78] Hoglinger D., Burgoyne T., Sanchez-Heras E., Hartwig P., Colaco A., Newton J., Futter C.E., Spiegel S., Platt F.M., Eden E.R. (2019). NPC1 regulates ER contacts with endocytic organelles to mediate cholesterol egress. Nat. Commun..

[79] Lim C.Y., Davis O.B., Shin H.R., Zhang J., Berdan C.A., Jiang X., Counihan J.L., Ory D.S., Nomura D.K., Zoncu R. (2019). ER-lysosome contacts enable cholesterol sensing by mTORC1 and drive aberrant growth signalling in Niemann-Pick type C. Nat. Cell Biol..

[80] Wijdeven R.H., Janssen H., Nahidiazar L., Janssen L., Jalink K., Berlin I., Neefjes J. (2016). Cholesterol and ORP1L-mediated ER contact sites control autophagosome transport and fusion with the endocytic pathway. Nat. Commun..

[81] Wang H., Ma Q., Qi Y., Dong J., Du X., Rae J., Wang J., Wu W.F., Brown A.J., Parton R.G., Wu J.W., Yang H. (2019). ORP2 delivers cholesterol to the plasma membrane in exchange for phosphatidylinositol 4, 5-bisphosphate (PI(4,5)P(2)). Mol. Cell.

[82] Meneses-Salas E., García-Melero A., Kanerva K., Blanco-Muñoz P., Morales-Paytuvi F., Bonjoch J., Casas J., Egert A., Beevi S.S., Jose J., Llorente-Cortés V., Rye K.A., Heeren J., Lu A., Pol A. (2020). Annexin A6 modulates TBC1D15/Rab7/StARD3 axis to control endosomal cholesterol export in NPC1 cells. Cell. Mol. Life Sci..

[83] Vihervaara T., Uronen R.L., Wohlfahrt G., Bjorkhem I., Ikonen E., Olkkonen V.M. (2011). Sterol binding by OSBP-related protein 1L regulates late endosome motility and function. Cell. Mol. Life Sci..

[84] Matsuo H., Chevallier J., Mayran N., Le Blanc I., Ferguson C., Faure J., Blanc N.S., Matile S., Dubochet J., Sadoul R., Parton R.G., Vilbois F., Gruenberg J. (2004). Role of LBPA and Alix in multivesicular liposome formation and endosome organization. Science.

[85] Neßlauer A.-M., Gläser A., Gräler M., Engelmann R., Müller-Hilke B., Frank M., Burstein C., Rolfs A., Neidhardt J., Wree A., Witt M., Bräuer A.U. (2019). A therapy with miglustat, 2-hydroxypropyl-ß-cyclodextrin and allopregnanolone restores splenic cholesterol homeostasis in Niemann-pick disease type C1. Lipids Health Dis..

[86] Maarup T.J., Chen A.H., Porter F.D., Farhat N.Y., Ory D.S., Sidhu R., Jiang X., Dickson P.I. (2015). Intrathecal 2-hydroxypropyl-beta-cyclodextrin in a single patient with Niemann-Pick C1. Mol. Genet. Metab..

[87] Ward S., O'Donnell P., Fernandez S., Vite C.H. (2010). 2-Hydroxypropyl-beta-cyclodextrin raises hearing threshold in normal cats and in cats with Niemann-Pick type C disease. Pediatr. Res..

[88] Vite C.H., Bagel J.H., Swain G.P., Prociuk M., Sikora T.U., Stein V.M., O'Donnell P., Ruane T., Ward S., Crooks A., Li S., Mauldin E., Stellar S., De Meulder M., Kao M.L. (2015). Intracisternal cyclodextrin prevents cerebellar dysfunction and Purkinje cell death in feline Niemann-Pick type C1 disease. Sci. Transl Med..

[89] Pipalia N.H., Subramanian K., Cross A., Garg K., Al-Motawa A., Maxfield F.R. (2019). Targeting molecular chaperone HSP90 to treat Niemann-Pick type C1 disease. FASEB J..

[90] Borbon I.A., Erickson R.P. (2011). Interactions of Npc1 and amyloid accumulation/deposition in the APP/PS1 mouse model of Alzheimer’s. J. Appl. Genet..

[91] Chan R.B., Oliveira T.G., Cortes E.P., Honig L.S., Duff K.E., Small S.A., Wenk M.R., Shui G., Di Paolo G. (2012). Comparative lipidomic analysis of mouse and human brain with Alzheimer disease. J. Biol. Chem..

[92] Park H., Kang J.H., Lee S. (2020). Autophagy in neurodegenerative diseases: A hunter for aggregates. Int. J. Mol. Sci..

[93] Carrière F., Longhi S., Record M. (2020). The endosomal lipid bis(monoacylglycero) phosphate as a potential key player in the mechanism of action of chloroquine against SARS-COV-2 and other enveloped viruses hijacking the endocytic pathway. Biochimie.

[94] Zaitseva E., Yang S.T., Melikov K., Pourmal S., Chernomordik L.V. (2010). Dengue virus ensures its fusion in late endosomes using compartment-specific lipids. PLoS Pathog..

[95] Pasqual G., Rojek J.M., Masin M., Chatton J.Y., Kunz S. (2011). Old world arenaviruses enter the host cell via the multivesicular body and depend on the endosomal sorting complex required for transport. PLoS Pathog..

[96] Chapuy-Regaud S., Subra C., Requena M., de Medina P., Amara S., Delton-Vandenbroucke I., Payre B., Cazabat M., Carriere F., Izopet J., Poirot M., Record M. (2013). Progesterone and a phospholipase inhibitor increase the endosomal bis(monoacylglycero)phosphate content and block HIV viral particle intercellular transmission. Biochimie.

[97] Moreau D., Vacca F., Vossio S., Scott C., Colaco A., Paz Montoya J., Ferguson C., Damme M., Moniatte M., Parton R.G., Platt F.M., Gruenberg J. (2019). Drug-induced increase in lysobisphosphatidic acid reduces the cholesterol overload in Niemann-Pick type C cells and mice. EMBO Rep..

[98] Dai S., Li R., Long Y., Titus S., Zhao J., Huang R., Xia M., Zheng W. (2016). One-step seeding of neural stem cells with vitronectin-supplemented medium for high-throughput screening assays. J. Biomol. Screen..

[99] Bedia C., Camacho L., Abad J.L., Fabrias G., Levade T. (2010). A simple fluorogenic method for determination of acid ceramidase activity and diagnosis of Farber disease. J. Lipid Res..

[100] Sun G., Yang K., Zhao Z., Guan S., Han X., Gross R.W. (2008). Matrix-assisted laser desorption/ionization time-of-flight mass spectrometric analysis of cellular glycerophospholipids enabled by multiplexed solvent dependent analyte-matrix interactions. Anal. Chem..

[101] Bligh E.G., Dyer W.J. (1959). A rapid method of total lipid extraction and purification. Can. J. Biochem. Physiol..

[102] Hsu F.F., Turk J., Shi Y., Groisman E.A. (2004). Characterization of acylphosphatidylglycerols from Salmonella typhimurium by tandem mass spectrometry with electrospray ionization. J. Am. Soc. Mass Spectrom..

[103] Guan Z., Li S., Smith D.C., Shaw W.A., Raetz C.R. (2007). Identification of N-acylphosphatidylserine molecules in eukaryotic cells. Biochemistry.

[104] McLoughlin H.S., Moore L.R., Chopra R., Komlo R., McKenzie M., Blumenstein K.G., Zhao H., Kordasiewicz H.B., Shakkottai V.G., Paulson H.L. (2018). Oligonucleotide therapy mitigates disease in spinocerebellar ataxia type 3 mice. Ann. Neurol..

