# Supplementary material for: Enrichment of NPC1-deficient cells with the lipid LBPA stimulates autophagy, improves lysosomal function, and reduces cholesterol storage
Source: J Biol Chem. 2021 May 21;297(1):100813. doi: 10.1016/j.jbc.2021.100813 (PMC8294588; doi:10.1016/j.jbc.2021.100813)
Supplement: Supplemental Figures S1–S4 [file mmc1.pdf]

Supplementary information

Enrichment of NPC1-deficient cells with the lipid LBPA stimulates autophagy, improves lysosomal function, and reduces cholesterol storage

Olga Ilnytska, Kimberly Lai, Kirill Gorshkov, Mark L. Schultz, Bruce Nguyen Tran, Maciej Jeziorek, Thaddeus J. Kunkel, Ruth D. Azaria, Hayley S. McLoughlin, Miriam Waghalter, Yang Xu, Michael Schlame, Nihal Altan-Bonnet, Wei Zheng, Andrew P. Lieberman, Radek Dobrowolski, Judith Storch

Supplemental Figures

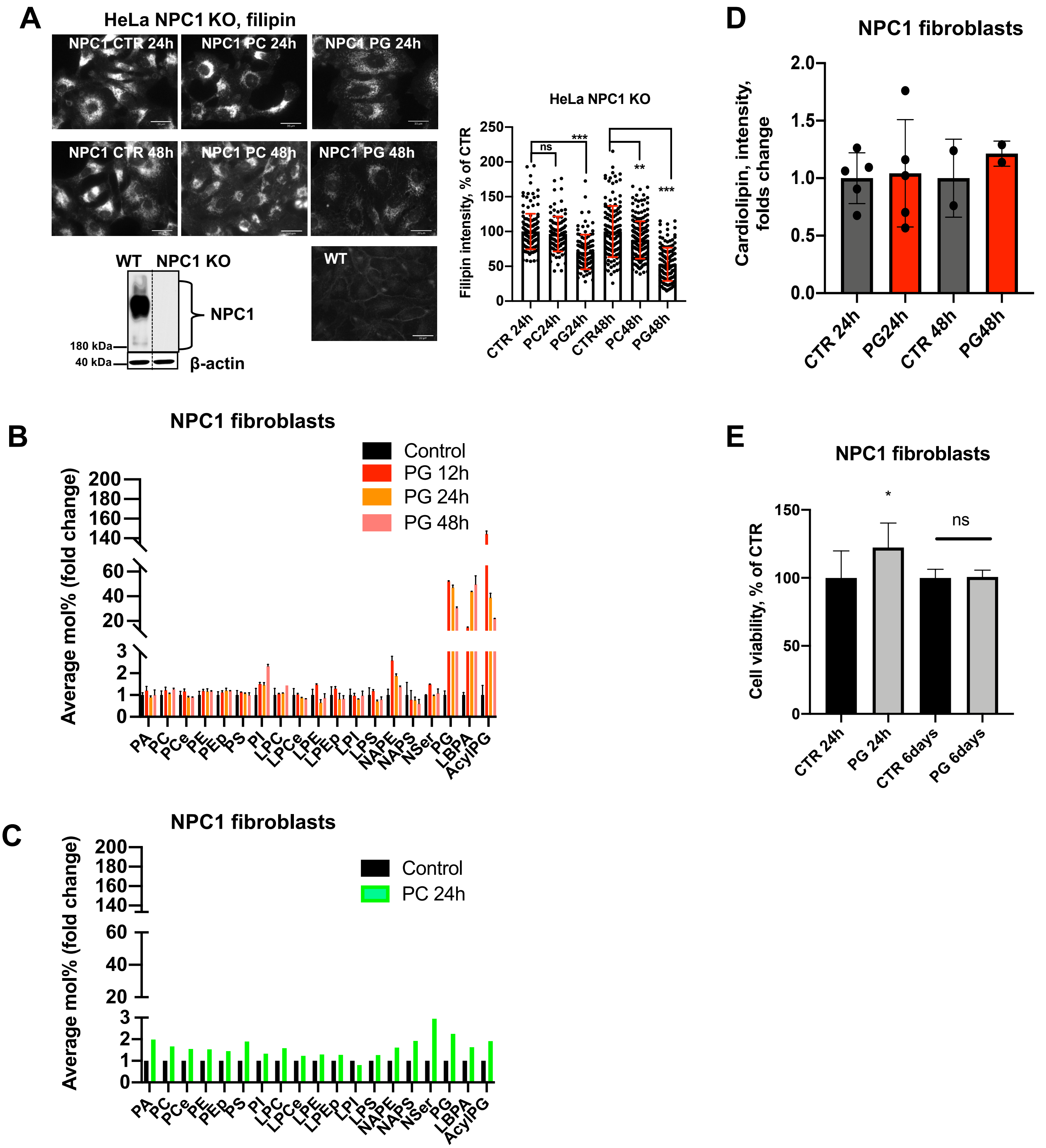

Fig. S1. Related to Fig. 1. Phospholipid composition of NPC1 mutant fibroblasts treated with PG. *A, r*epresentative filipin images and quantification of filipin intensity in parental (WT) and PG or PC treated CRISPR-Cas9 edited NPC1 KO HeLa cells. Western blot demonstrates stable NPC1 gene deletion. In Western Blot samples were analyzed in biological replicates. For each condition one band was cropped and bands for WT and NPC1 KO were spliced. Splicing is indicated with a vertical line. In images, bar=30µm. Data from 2 independent experiments. N=100-200 cells/condition. *B,* LC-MS analysis of NPC1-mutant fibroblasts treated with 100µM PG for 12, 24 and 48h. Bar graph indicate fold-change of average relative mol% of all lipids measured, normalized to untreated CTR. N=2. *C,* LC-MS analysis of NPC1-mutant fibroblasts treated with 100µM PC for 24h. Bar graph indicate fold-change of average relative mol% of all lipids measured, normalized to CTR. *D,* LC-MS analysis of cardiolipin in NPC1-deficient fibroblasts treated with 100µM PG for 24 and 48h relative to untreated CTR. *E,* Cell viability assay in NPC1 deficient cells treated with PG. All graphed data show mean ±SD. *p<0.05, **:p<0.01, ***:p<0.001 compared to untreated cells (CTR) in two-tailed Student's test.

Nomenclature abbreviations are: PA, phosphatidic acid; PC, phosphatidylcholine; PCe, ether phosphatidylcholine; PE, phosphatidylethanolamine; PEp, plasmalogen phosphatidylethanolamine; PG, phosphatidylglycerol; PI, phosphatidylinositol; PS, phosphatidylserine; LPC, lysophosphatidylcholine; LPCe, ether lysophosphatidylcholine; LPE, lysophosphatidylethanolamine; LPEp, plasmalogen lysophosphatidylethanolamine; LPI, lysophosphatidylinositol; LBPA lysobisphosphatidic acid; AcylPG acyl phosphatidylglycerol, NAPE N-acyl phosphatydylethanonamine, NAPS N-acyl-phosphatidylserine, NSer N-acylserine

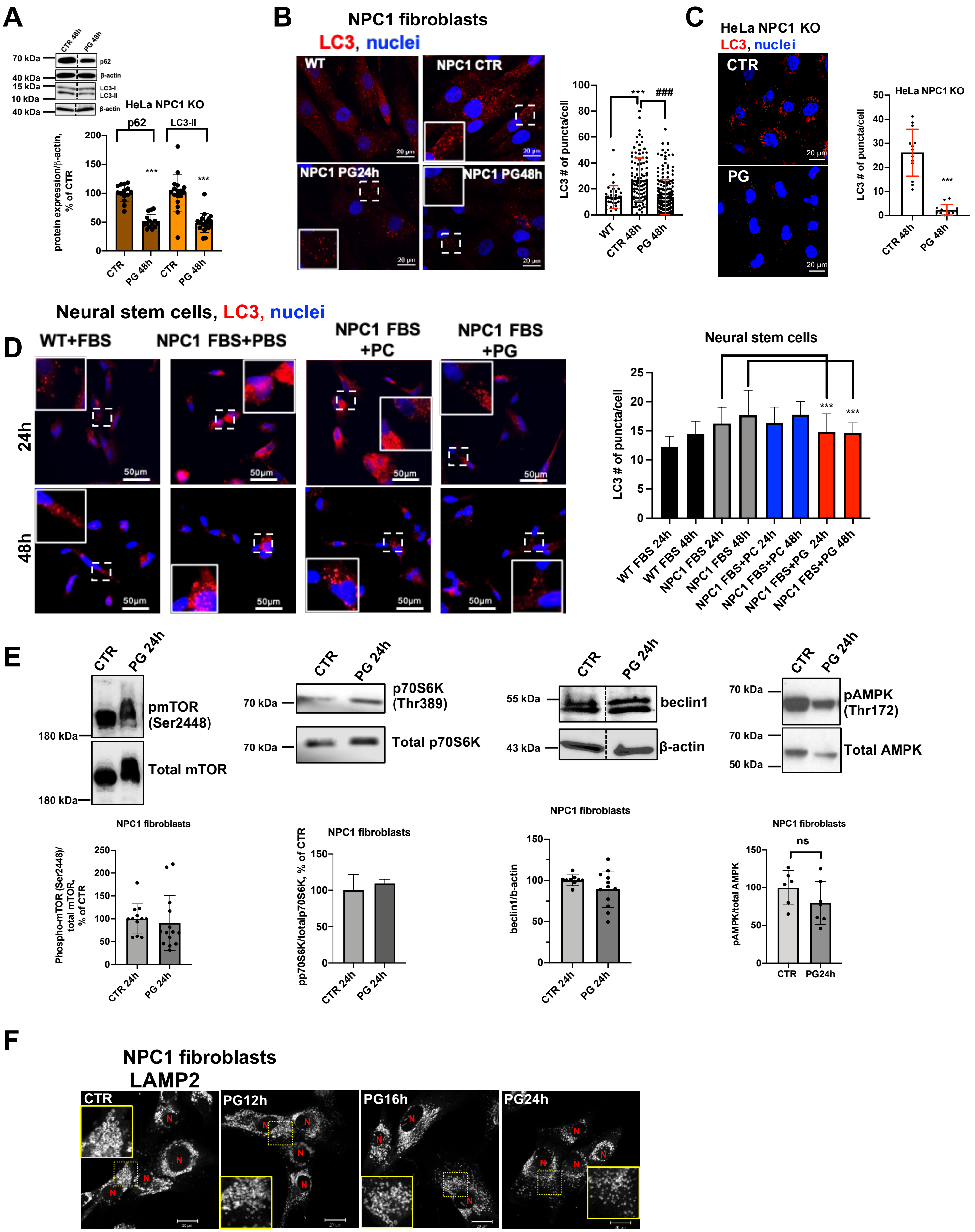

A
CTR 48h
PG 48h
70 kDa
p62
40 kDa
β-actin
15 kDa
LC3-I
LC3-II
10 kDa
40 kDa
β-actin
HeLa NPC1 KO
p62
LC3-II
protein expression/β-actin, % of CTR
200
150
100
50
0
***
***
CTR
PG 48h
CTR
PG 48h
B
NPC1 fibroblasts
LC3, nuclei
WT
NPC1 CTR
NPC1 PG24h
NPC1 PG48h
20 µm
LC3 # of puncta/cell
100
80
60
40
20
0
***
###
WT
CTR 48h
PG 48h
C
HeLa NPC1 KO
LC3, nuclei
CTR
PG
20 µm
HeLa NPC1 KO
LC3 # of puncta/cell
50
40
30
20
10
0
***
CTR 48h
PG 48h
Neural stem cells, LC3, nuclei
D
WT+FBS
NPC1 FBS+PBS
NPC1 FBS +PC
NPC1 FBS +PG
24h
48h
50µm
Neural stem cells
LC3 # of puncta/cell
25
20
15
10
5
0
***
***
WT FBS 24h
WT FBS 48h
NPC1 FBS 24h
NPC1 FBS 48h
NPC1 FBS+PC 24h
NPC1 FBS+PC 48h
NPC1 FBS+PG 24h
NPC1 FBS+PG 48h
E
CTR
PG 24h
pmTOR (Ser2448)
180 kDa
Total mTOR
180 kDa
70 kDa
p70S6K (Thr389)
70 kDa
Total p70S6K
55 kDa
beclin1
43 kDa
β-actin
70 kDa
pAMPK (Thr172)
70 kDa
Total AMPK
50 kDa
NPC1 fibroblasts
Phospho-mTOR (Ser2448)/ total mTOR, % of CTR
250
200
150
100
50
0
CTR 24h
PG 24h
NPC1 fibroblasts
pp70S6K/totalp70S6K, % of CTR
150
100
50
0
CTR 24h
PG 24h
NPC1 fibroblasts
beclin1/b-actin
150
100
50
0
CTR 24h
PG 24h
NPC1 fibroblasts
ns
pAMPK/total AMPK
150
100
50
0
CTR
PG24h
F
NPC1 fibroblasts
LAMP2
CTR
PG12h
PG16h
PG24h
N

Fig. S2. Related to Fig. 2 and 3. PG treatment does not alter early autophagic markers in NPC1-deficient fibroblasts

A, Western Blot analysis and quantification of LC3-II and p62 in control and PG-treated NPC1 KO HeLa cells at 48h post-treatment. Data from 3-5 independent experiments. On the upper panel samples were analyzed in biological replicates. For each condition one band was cropped and bands for CTR and PG were spliced. Splicing is indicated with a vertical line. *B,* Representative images and quantification of LC3-positive puncta in NPC1 fibroblasts at 48h. Scale bar, 20µm. N>60 cells/condition. *C,* Representative confocal images and quantification of LC3 staining in control and PG-treated NPC1 KO HeLa cells at 48h post-treatment. *D,* Representative images and quantification of LC3 puncta in neural stem cells treated with 50µM PC or PG liposomes. *E,* Western Blot analysis and quantification of autophagy markers in NPC1-deficient fibroblasts. Splicing is indicated with a vertical line. *F,* Time course of labeled with LAMP2 LY distribution in NPC1 deficient fibroblasts treated with PG. Insets illustrate the size of LY. N-nuclei. Scale bar, 20µm. All graphed data show mean ±SD. ***:p<0.001 compared to untreated cells (CTR) in two-tailed t-test. Error bars indicate SD.

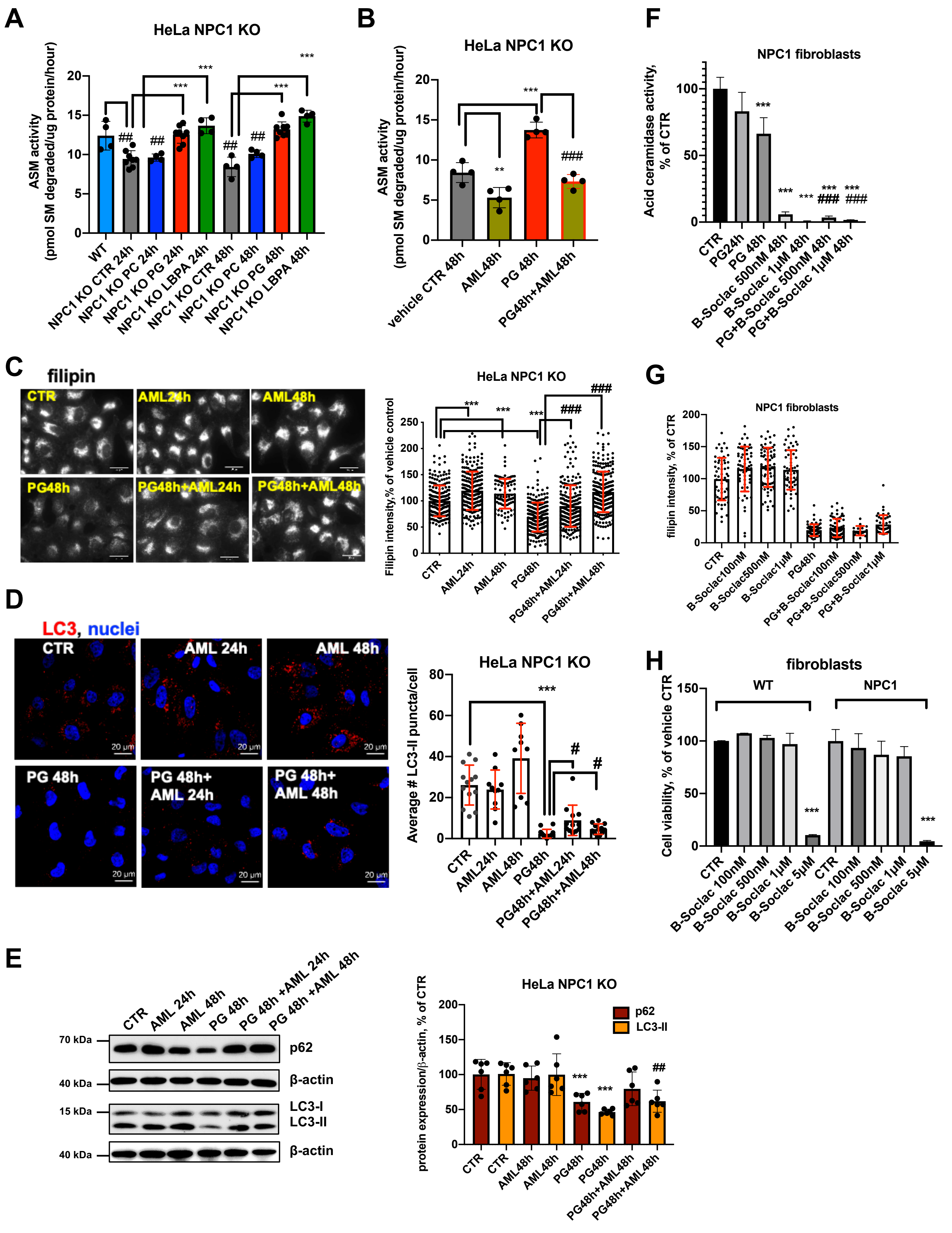
A
HeLa NPC1 KO
ASM activity
(pmol SM degraded/ug protein/hour)
WT
NPC1 KO CTR 24h
NPC1 KO PC 24h
NPC1 KO PG 24h
NPC1 KO LBPA 24h
NPC1 KO CTR 48h
NPC1 KO PC 48h
NPC1 KO PG 48h
NPC1 KO LBPA 48h
B
HeLa NPC1 KO
ASM activity
(pmol SM degraded/ug protein/hour)
vehicle CTR 48h
AML48h
PG 48h
PG48h+AML48h
F
NPC1 fibroblasts
Acid ceramidase activity,
% of CTR
CTR
PG24h
PG 48h
B-Soclac 500nM 48h
B-Soclac 1μM 48h
PG+B-Soclac 500nM 48h
PG+B-Soclac 1μM 48h
C
filipin
CTR
AML24h
AML48h
PG48h
PG48h+AML24h
PG48h+AML48h
HeLa NPC1 KO
Filipin intensity,% of vehicle control
G
NPC1 fibroblasts
filipin intensity, % of CTR
CTR
B-Soclac100nM
B-Soclac500nM
B-Soclac1μM
PG48h
PG+B-Soclac100nM
PG+B-Soclac500nM
PG+B-Soclac1μM
D
LC3, nuclei
CTR
AML 24h
AML 48h
PG 48h
PG 48h+
AML 24h
PG 48h+
AML 48h
20 μm
HeLa NPC1 KO
Average # LC3-II puncta/cell
CTR
AML24h
AML48h
PG48h
PG48h+AML24h
PG48h+AML48h
H
fibroblasts
Cell viability, % of vehicle CTR
WT
NPC1
CTR
B-Soclac 100nM
B-Soclac 500nM
B-Soclac 1μM
B-Soclac 5μM
E
CTR
AML 24h
AML 48h
PG 48h
PG 48h +AML 24h
PG 48h +AML 48h
70 kDa
40 kDa
15 kDa
40 kDa
p62
β-actin
LC3-I
LC3-II
β-actin
HeLa NPC1 KO
protein expression/β-actin, % of CTR
p62
LC3-II
CTR
AML48h
PG48h
PG48h+AML48h

Fig. S3. Related to Fig. 4. ASM inhibition reverses the effects of PG/LBPA administration in NPC1-knockout cells.

A, ASM activity in parental and NPC1 KO HeLa cells treated with anionic phospholipids for 24 and 48h: PG (100µM), PC (100µM) and PC:LBPA (1:1 mol:mol) (100µM:100µM). ***p<0.001 vs NPC1 KO control. ##p<0.01 vs parental cell line (WT HeLa). *B, e*fficient inhibition of ASM in AML treated NPC1 KO HeLa cells without and with 100µM PG treatment. AML concentration was 5µM. AML was added alone or together with PG for 48h. *C,* Representative epifluorescent images of filipin staining in NPC1 KO HeLa cells treated with DMSO (48h), AML alone (24 and 48h), PG alone (48h) or in combination of PG (48h) and AML (24 and 48h) and quantification. Filipin intensity was quantified in >50 cells in each group and data represented as % of DMSO control. Scale bar, 30µm. Statistically significant changes are indicated. ***p<0.001 vs DMSO control. ###p<0.001 vs PG alone in two-tailed Student's test. AML concentration 5µM. *D***,** representative confocal images and image quantification of LC3-II in NPC1 KO HeLa cells treated with DMSO, AML, PG, or PG+AML at indicated time points as in C. LC3-II-positive puncta were quantified by intracellular staining using a-LC3 antibody and represented as # per cell. Individual dots represent average # per cell/image. Average cell # in image is 8 cells. Scale bar is 20 µm. ***:p<0.001 compared to DMSO control, #:p<0.05, in two-tailed Student's test. Error bars indicate SD. AML concentration 5µM, PG 100µM. *E,* Western blot and quantification of LC3-II and p62 autophagy markers in NPC1-deficient fibroblasts treated with DMSO, AML, PG, PG+AML as in C and D. β-actin was used as loading control. AML 5µM, PG 100µM. *F,* Acid ceramidase activity in NPC1 deficient fibroblasts treated with 100µM PG and 500nM or 1µM of the acid ceramidase inhibitor Bodipy-Soclac. Inhibitor was added alone or together with PG for 48h. *G,* Filipin intensity quantification in NPC1-deficient fibroblasts treated with PG (100µM) and indicated concentrations of Bodipy-Soclac. Bodipy-Soclac was added alone or together with PG for 48h. *H,* Cell viability assay for 48h treatment of WT and NPC1 fibroblasts with Bodipy-Soclac in concentration range 100nM-5µM. All graphical data expressed as mean ± SD.

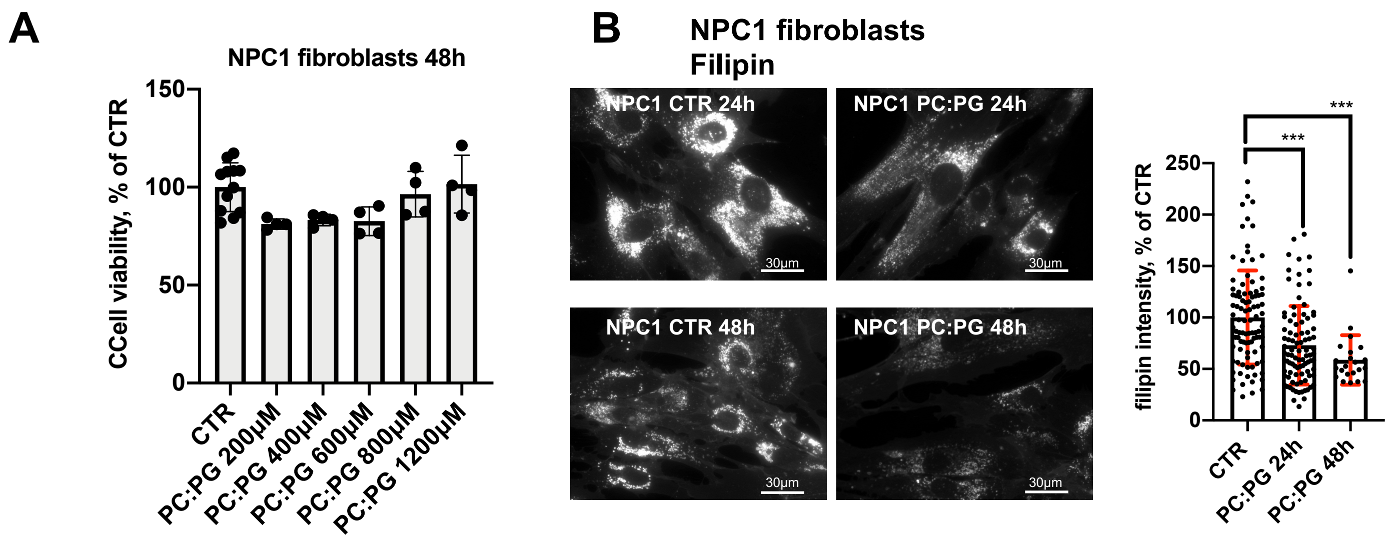

Fig. S4. Related to Figure 6. Cell viability and filipin imaging following treatment with PC:PG. *A,* cell viability following treatment with PC:PG at 48h. Cell viability was determined using Cell Titer-Glo assay. The concentration of total lipid is presented: e.g. 200µM PC:PG (1:1 mol:mol) (100µM PC+100µM PG). *B, r*epresentative images and filipin quantification in PC:PG treated NPC1 fibroblasts at 24 and 48h. Scale bar, 30µm. Bar graph represents data from 2 independent experiments. ***:$p<0.001$ compared to untreated cells (CTR) in two-tailed t-test. 200µM PC:PG (1:1 mol:mol) (100µM PC+100µM PG).
All graphed data show mean ±SD.
